# Public communication by research institutes compared across countries and sciences: Building capacity for engagement or competing for visibility?

**DOI:** 10.1371/journal.pone.0235191

**Published:** 2020-07-08

**Authors:** Marta Entradas, Martin W. Bauer, Colm O'Muircheartaigh, Frank Marcinkowski, Asako Okamura, Giuseppe Pellegrini, John Besley, Luisa Massarani, Pedro Russo, Anthony Dudo, Barbara Saracino, Carla Silva, Kei Kano, Luis Amorim, Massimiano Bucchi, Ahmet Suerdem, Tatsuo Oyama, Yuh-Yuh Li

**Affiliations:** 1 Instituto Universitário de Lisboa (ISCTE-IUL), Lisbon, Portugal; 2 Department of Psychological and Behavioural Science, London School of Economics and Political Science, London, England, United Kingdom; 3 University of Chicago Harris School of Public Policy, Chicago, Illinois, United States of America; 4 Department of Social Sciences, Heinrich-Heine-Universität Düsseldorf, Düsseldorf, Germany; 5 National Graduate Institute for Policy Studies, Tokyo, Japan; 6 Observa Science & Society, Vicenza, Italy; 7 Department of Advertising and Public Relations, Michigan State University, East Lansing, Michigan, United States of America; 8 Fundação Oswaldo Cruz, FIOCRUZ, Casa de Oswaldo Cruz, Instituto Nacional de Comunicação Publica da Ciência e Tecnologia, Rio de Janeiro, Brazil; 9 Department of Science Communication & Society, Leiden University, Leiden, The Netherlands; 10 Stan Richards School of Advertising and Public Relations, University of Texas at Austin, Austin, Texas, United States of America; 11 Department of Sociology, Università di Trento, Trento, Italy; 12 Faculty of Education, Shiga University, Otsu Shiga, Japan; 13 Department of Political and Social Sciences, University of Bologna, Bologna, Italy; 14 Bilgi University, Istanbul, Turkey; 15 National Sun Yat-sen University, Kaohsiung, Taiwan; IUMPA - Universitat Politecnica de Valencia, SPAIN

## Abstract

Leading academic institutions, governments, and funders of research across the world have spent the last few decades fretting publicly about the need for scientists and research organisations to engage more widely with the public and be open about their research. While a global literature asserts that public communication has changed from a virtue to a duty for scientists in many countries and disciplines, our knowledge about what research institutions are doing and what factors drive their ‘going public’ is very limited. Here we present the first cross-national study of N = 2,030 research institutes within universities and large scientific organisations in Brazil, Germany, Italy, Japan, the Netherlands, Portugal, the United Kingdom, and the United States of America. We find that institutes embrace communication with non-peers and do so through a variety of public events and traditional news media–less so through new media channels–and we find variation across countries and sciences, yet these are less evident than we expected. Country and disciplinary cultures contribute to the level of this communication, as do the resources that institutes make available for the effort; institutes with professionalised staff show higher activity online. Future research should examine whether a real change in the organisational culture is happening or whether this activity and resource allocation is merely a means to increase institutional visibility.

## Introduction

In recent decades, we have witnessed a growing tendency within academic and research organisations to open doors and turn to the broader public [1] [2] [3]. Institutions have extended their audiences to include students, funders of research, journalists, NGOs, business and industry, and various segments of the public, even counter publics [4], and have increased the panoply of formats of communication activities directed to these groups [1].

This change towards broader public communication is not entirely new–indeed outreach and community engagement have been the ‘third mission’ of most universities around the world [5]. Yet, it is likely that this revival has assumed salience for different purposes: with developments in academic research assessments, funded research is required to facilitate ‘pathways to impact’ on society [6] [7]. This has brought pressure on institutions to open up to public communications, and to compete for public visibility [8] [9] [10], which is likely to have consequences for the societal conversation around science. Hitherto, the empirical research on this communication activity of research organisations has been scattered and not on a comparable basis.

The few systematic studies that have shed light onto organisational public communication focused on the functions of central PR offices of universities [3] [11] [12] [13] and large European research organisations [14] for media science communication. These studies point to a growing orientation on the part of organisations towards the media and journalists to build a reputation and image through self-promoting scientific findings and scientists. For example, Marcinkowski & Kohring (2014) [3] found that the central communications offices in German universities tend to instrumentalise the vanity of many scientists who like to be in the media, to build institutional reputation in the public sphere rather than scientists’ career interests. At this central level, communication about research findings acquires a special selectivity: research that serves news values well (surprise, large numbers, crises and catastrophes, conflict, human interest, etc.) has a better chance of being communicated than research that contributes to enlightening society [8]. From these findings it seems evident that at the central communications level, communication of science serves the goals of public visibility rather than public engagement.

We know less about public communication at other levels of the scientific organisation. For example, little is known about the decentralised communication structures and functions at the level of research centres, institutes, research units (under various names across national research systems), that are more likely to be in a position to communicate about their area of study–what for a better term, we call the meso-level of research institutes [15]. This level of investigation contrasts with studies of the central communication offices of universities, and the individual-scientist-level. The lack of research focused on this level is surprising given the role of research institutes in building science-society relations. Not only they are the places where scientific knowledge is produced, but they also have a privileged position in accessing publics and influencing public debate.

Despite this, there have been preliminary attempts to measure communication activities of research institutes in Portuguese universities and research organisations [1]. This study reports emerging capacity building measures for public communication of the institutes’ research activities, with public events and media interactions led independently from the central university PR offices and locally resourced [1] [15]. This work has however been recorded at the national level. Little is known about how the activity compares across institutes in different countries, with distinct traditions of public engagement in science [16] [17], scientific systems, and R&D resources [18], that allows for a broader understanding of this capacity building in contexts of internationally increasing public engagement demands [19] [20] [6].

In 2018, we conducted a systematic multi-country study of research institutes with a twofold goal: to comparatively assess public communication across countries and areas of research, and to examine the factors that explain public communication activity. We investigated a stratified sample of N = 2,030 institutes in Brazil, Germany, Italy, Japan, the Netherlands, Portugal, the United Kingdom, and the United States of America, covering the categories of the Organisation for Economic Cooperation and Development (OECD) including the Natural Sciences, the Engineering and Technology, the Medical and Health Sciences, Agriculture, the Social Sciences and the Humanities [21].

We test the hypotheses that public communication by research institutes varies across countries and sciences, and that the context in which the research institute is embedded and the institute’s commitment to communication are further conditions for such activity. We build our analytic framework on models of public communication of individual scientists, and preliminary findings on institutional drivers of communication. This research has pointed to disciplinary cultures in science communication, both in terms of the intensity and the choices of formats of engagement. Natural sciences have often been found to be less active than social sciences [22] [23] [24]–astronomy [25], and climate science [26] may be exceptions. Natural sciences engage more in educational activities for schools and the wider public; social sciences tend to be more active in civic-related activities [1]. Yet, the question relating to how science communication varies across countries and global regions has received very little attention. The handful of cross-country comparisons point to some country variation in science communication [27] [16] [28] [25], but these variations are small among scientists. More recent research shows that this activity associates with the organisational contexts in which scientists work: our global survey of astronomers (N = 2,600) showed that those scientists working with more resources from their institutions were also more active communicators, regardless of the global region in which they worked [25].

With limited comparative data on the organisational side, it is not our goal here to frame working hypotheses as to which countries or disciplines have higher activity. Our goal is to offer first empirical observations as a baseline study on cultures of science communication across countries and disciplines at the level of research institutes, to define key concepts and to operationalise key indicators of public communication of research institutes. Exploring differences and similarities across countries and fields will allow for future research and discussion aimed at understanding any patterns found by the current study.

In our analyses, we distinguish between three different formats of communication–public events-making (public events hereafter), traditional news media access (traditional media), and the use of new media channels (new media); they differ in nature and require different resources, which may influence the choices of communication that are adopted by organisations. That is, we would expect variation in the use of these formats, and this variation to be associated with the mobilisation of resources within the institution. These distinctions allow for a better understanding of the portfolio of formats and the outcomes that can be achieved.

Against this backdrop, our conceptual framework examines public communication as a function of the general organisational context and organisations’ dispositions to communicate publicly. That is, the level of public communication activity of an institute (P) can be explained by the combination of the general context of the organisation (C factors) and disposition factors that characterise the specific orientation towards communication activities (D factors) (P = *f* (C, D). C factors reflect the features of the institution and research environment, such as the country and scientific discipline, and the size of their annual research budget–factors known to influence communications [1]. D factors reflect the commitment and responsibility at the level of institutes that encourages public communication and supports the development of such activities; it is operationalised by the available funding for communications (research budget), guidance by communication policies (policy), recruitment of professional communications staff (staffing), and the degree to which research scientists are involved (active researchers). We know that researchers engage in public communication, but we do not know their contribution to the overall communication effort of a research institute. Similarly, explicit policies that encourage science-society relations have been adopted in many countries and institutions, and the impact of policy on practices of public communication in a country has been debated [29]. Here we test whether such communication policies are in place on the ground of research institutes and the impact they might have in the level of activity.

This framework builds on a model that we previously used to understand individual scientists’ communication activity [26] derived from Lewin’s field-theory model of behaviour [30]. It considers behaviour to be situated by factors both internal (dispositions) and external (context) to the unit of analysis. We build on this idea and extend the model to research institutes–our unit of analysis–considering their context and their commitment to public engagement as two sets of factors affecting public communication. This framework combines factors often correlated with public communication: sociological indicators of context, and social-psychological indicators of commitment. Our framework helps to understand these relationships by comparing the effects of these two sets of factors and investigating how they behave when together in the same models. That is, how much of the variation in the level of public communication of an institute is derived from country or discipline cultures, and how much from factors inherent to the research organisation. For example, while we expect resources to play an important role, it is also possible that institutes with higher levels of resources may not show a higher level of communication activity because other factors play a stronger role. Importantly, it helps to distinguish these two sets of conditions of public communication and will offer insights into the portfolio choices of institutes in their communication. In this study, we used the term ‘public communication’–adapted to the various national contexts–to refer broadly to any type of communications activities with external audiences.

## Methods and data

### Procedure and sample design

An online survey was distributed to research institutes in the surveyed countries between June 2017 and May 2018. Data was centrally collected by the principal investigator (PI) with a questionnaire implemented on Qualtrics software translated into local languages. In Portugal data had been collected earlier in 2015 as a pilot study. Institutional culture shifts are unlikely to happen in short periods of time, we therefore assume data comparability is preserved.

Two weeks before the questionnaires were distributed, respondents were pre-notified by email about the study and asked for collaboration. This first contact also served to refine the samples. We asked institutes to confirm that they were a research-active institute. For each institute we targeted one respondent, addressed by name and title, and name of the institute; this was the person most likely in a position to assess the communication efforts of the institute, either a responsible for communications tasks or the director/head of the institute. We could not control for the respondents’ role as many of these institutes are small and do not have a communications person [1]; in those cases we addressed the directors. In our study, 41% of respondents were Directors/Heads/Coordinators, 17% were management/administrative staff, 13% were communications staff, and 22% were researchers; 18% of the questionnaires were answered by two or more people in collaboration. All respondents reported to be highly involved in the institute’s communications activities and values.

We used Entradas and Bauer’s (2017) measurement instrument for comparability, with questions on practices, rationales, resources for public communications, and stereotypical perceptions of the public. Here we report data on communication practices and their resourcing. The questionnaire was developed in English and translated into German, Italian, Japanese, and Brazilian Portuguese, and back translated into English [31] for quality control. Questionnaires were piloted among communications staff in all countries and final adjustments were made, whenever needed. One questionnaire was collected per institute. Questionnaires complete to less than 70% were discarded.

#### Mixed methods to boost response rates

A number of modes were used to collect data. Given the intensifying challenge of obtaining responses to surveys, this is now standard practice in survey methodology [e.g. [32]]. There is strong evidence that the between-mode differences are slight compared to the potential bias of lower response rates (RRs).

An average of three reminders to the web surveys were sent per country. National teams made additional efforts, according to the resources available. In Portugal, Italy, the UK, and Netherlands, non-respondents were contacted by telephone and encouraged to participate. This resulted in an increase between 10% - 20% in the total RR. In the UK and the USA, a mail survey was conducted with a subsample of non-respondents (N = 150) (see S1 Table). The questionnaire was mailed together with an addressed envelope to return the papers, and reminder postcards were mailed two weeks later. This method resulted in a further increase of 10% in the total RR in both countries.

### Sample design

We built sampling frames of research institutes from official lists from governments and/or funding bodies when they were available (Netherlands, Portugal, Italy), and from universities’ websites in countries where lists of research institutes were not available (the UK, the USA, Japan, and Brazil). Entire populations of institutes were included in countries with smaller number of institutes as in the Netherlands, Italy, Portugal and Japan. In the countries where mapping was not complete (UK, USA, Brazil, and Germany) we built sampling frames from a structured set of universities and mapped all research institutes within them. Each institute listed was classified according to OECD scientific areas [21] our primary stratification variable. We used disproportionate stratified probability sampling to generate representative samples of the institute populations, accounting for areas of research. For the population of research institutes as defined in each country’s plan, every research institute was included in the sampling frame for the country and had a known non-zero probability of selection. Within each stratum (scientific area) an independent sample of research institutes was selected. We aimed at an N = 200 institutes per stratum–agreed by the investigator team as a group–resulting in a total sample of around N = 1,200, except for countries where populations had less cases. A sample of size 200 would, *ceteris paribus*, produce an estimate of a proportion (percentage) with a standard error less than or equal to + or—0.036 (3.6%) and a confidence interval of + or—0.07 (7%). This would provide a satisfactory basis for inclusion of a country’s results in the analysis.

Disproportionate stratified sampling was preferred to ensure that an adequate number of sampled research institutes was included from each of the strata (scientific areas). Using proportionate stratification (same sampling fraction in each stratum) would lead to inadequate numbers for analysis in scientific areas with smaller numbers of institutes, and make analysis by scientific area less precise. In generating population estimates, the differences in sampling fraction are taken into account in the estimation through appropriate weighting. S1 Table describes in detail the country sampling procedures.

N = 8033 was the total number of research institutes approached overall. S2 Table presents the distribution across countries and scientific areas, and RRs. A total of N = 2,030 institutes responded in our survey. The overall weighted response rate (WRR) was 25%. Weights were calculated for each unit to compensate for differential probabilities of selection and response rates among stratum [33]. We report both weighted and unweighted RR because probabilities of selection are not equal for all elements, thus both rates should be presented. While this rate may seem low, this level is expected for on-line web surveys [34]^,^[35], which hardly reach 25% without massive reinforcement and reminding. Similarly, for industry unit level surveys of universities, academies and corporate business units, 25% is a very realistic and good response rate.

The final samples are unbiased across research areas; there were no significant differences in the institutions contacted (target samples) and institutions that responded (samples) per country and (χ2 *p* > .05) (see S1 Text).

### Dependent variables

Our three dependent variables measured the level of public communication activity: “public event making”, “traditional news media”, and “new media channels”. We asked respondents to report estimated frequencies of activities in the past 12 months prior to the survey. Activities were measured on an ordinal scale: Never (none); Annually (once a year), Quarterly (2–6 times a year), Monthly (7–20 times a year), Weekly, ‘Don’t know’. For new media channels with a shorter natural cycle of activities, we added the option ‘Daily’ and dropped the option ‘Once a year’. “Public event making” (9 items) included public lectures, public exhibitions, open days, science festivals/fairs, science cafés/debates, policy-making events, workshops with private organisations, talks at schools, and citizen science projects; “Traditional news media” (13 items) included interviews for newspapers, interviews for the radio, interviews for the TV, other TV, press conferences, press releases, newsletters, brochures/non-academic publications, articles in magazines, multimedia, popular books, policy briefs, materials for schools; “New media” (6 items) included website (updates), blogs, Facebook, Twitter, Youtube, and Podcasts.

#### Indices of public communication activity

We built two types of indicators of intensity of public communication activity for the purpose of analysis:

- Indices from estimated number of activities. Construction of these indices was based on the frequency of engagement reported by institutes and derived by recoding variables in the number of participations. Scales were re-coded to median frequency estimates as follows: never (0), annually (1), quarterly (4), monthly (12), weekly (48; referring to the number of work weeks per year) and daily (240; for 48 work weeks per year). Reliability analysis shows high internal consistency (Cronbach’s α = .70 for public events, .85 for traditional news media and .71 for new media) (S3 Table).
- Indices from factor scores. We used categorical confirmatory factor analysis (CFA) to build indices of intensity for events, channels and new media. These three dimensions were identified in a preliminary study (1), and here we confirm these with robust data form various countries. (S4 Table).

Given the skewedness of the data, we recoded each event and traditional channel into ‘never’ (0), ‘once a year’ (1), and ‘more than once a year’ (2); and ‘no’ (0) and ‘yes’ (1) referring to use/no use of new media. The CFA resulted in a scale structure with a strong internal consistency with items loading appropriately for public events, traditional channels and social media. The model fitted reasonably well, but we refined the scales by taking out items with loadings below 0.30 (policy-making events, workshops with private organisations, newsletters, brochures/non-academic publications, multimedia, website) (standardised loadings ranged from 0.152 to 0.482). Model fits for the refined model are χ2 = 627.54, CFI = 0.96, RMSEA = 0.04, TLI = 0.95, BIC = 56474.49, df = 142, p<0.001) (S4 Table). Higher scores indicate higher levels of activity.

### Independent variables

#### C variables: The organisational environment

We controlled for the effect of the size of the institution, which is likely to have an influence in level of public communication activity; and for the effect of research budget given the well know the varying distribution of national research budgets in different countries and disciplines.

*Size* is a count variable for the number of researchers working at the institute. This variable was recoded as (1) less than 20 researchers), (2) 20–80 researchers, (3) 80 researchers or more.

*Annual Research budget* is a categorical variable measured at the ordinal level: (1) less than €100.000, (2) €100.000- €250.000 euros, (3) €250.000–500.000, (4) €500.000-€1M, and (5) more than €1M. Amounts were converted from the country’s local currency.

*Country* is recoded into 8 dichotomous variables; the agriculture (highest level of activity) is the reference category. *Research area* is recoded into 6 dichotomous variables; Brazil is the reference category.

#### D variables: Dispositions to communicate publicly

*Active researchers*. To measure the level of researchers’ involvement, we asked institutes what “percentage of researchers [in your research institute] engaged in public communication activities in the past 12 months?” This was an ordinal variable coded (1) for none, (2) less than 10%, (3) 10%-20%, (4) between 20%-40%, (5) 40%-60%, (6) 60–100%.

*Communications policy*. We asked institutes whether they had a policy in place for public communication; ‘policy’ was a binary variable coded (1) yes, (0) no. About 48% of the institutes reported having a policy in place for public communication.

*Communications staff*. We asked institutes whether they employed specialist staff dedicated to public communication tasks. Options were (1) we have staff ‘within the institute’, (2) ‘we do not have staff within the institute but have access to the central level/PR office of the institution/university’, and (3) ‘none’. This variable was recoded into a binary with (1) for’ staff within the unit’, and (0) ‘no local staff’ (this combined original options 2 and 3), because we wanted to distinguish between those institutes that employ staff and those that do not. In 38% of institutes there are specialist communications staff; M = 2 to 3 FTE staff among institutes that reported employing communications staff.

*Communications funding*. We asked institutes “how much of your annual budget have you allocated in the past 12 months to public communication activities? Please do not consider salaries of ‘communication staff’.” This was an ordinal estimate coded (1) none, <1% (coded 2), 1–5% (coded 3), 5–10% (coded 4), >10% (coded 5). About 52% reported spending < = 1%.

See S5 Table for distribution of responses by variables.

### Statistical analysis

We used one-way ANOVAs to compare across groups (S6 Table) and pairwise Bonferroni post hoc tests to determine which groups differed significantly from each other. We use two-step hierarchical regression models and investigate the effects of the two sets of variables. Country and disciplinary cultures are likely to have an effect on public communication, in addition to institutes’ characteristics such as size and research budget, inserted in step 1, Model 1). In step 2, we determined whether D factors showed a significant improvement in the proportion of explained variance in our dependent variables (Model 2). We compare the effect of the predictors by comparing the standardised regression coefficients. We report the adjusted R^2^, R^2^ Change, and F values, Unstandardised coefficients, and Standardised Beta values, and p values (S7–S9 Tables). We used an alpha level of .05 for all statistical tests. All models were significant and explained a substantive amount of the variance.

## Results

**Fig 1:**
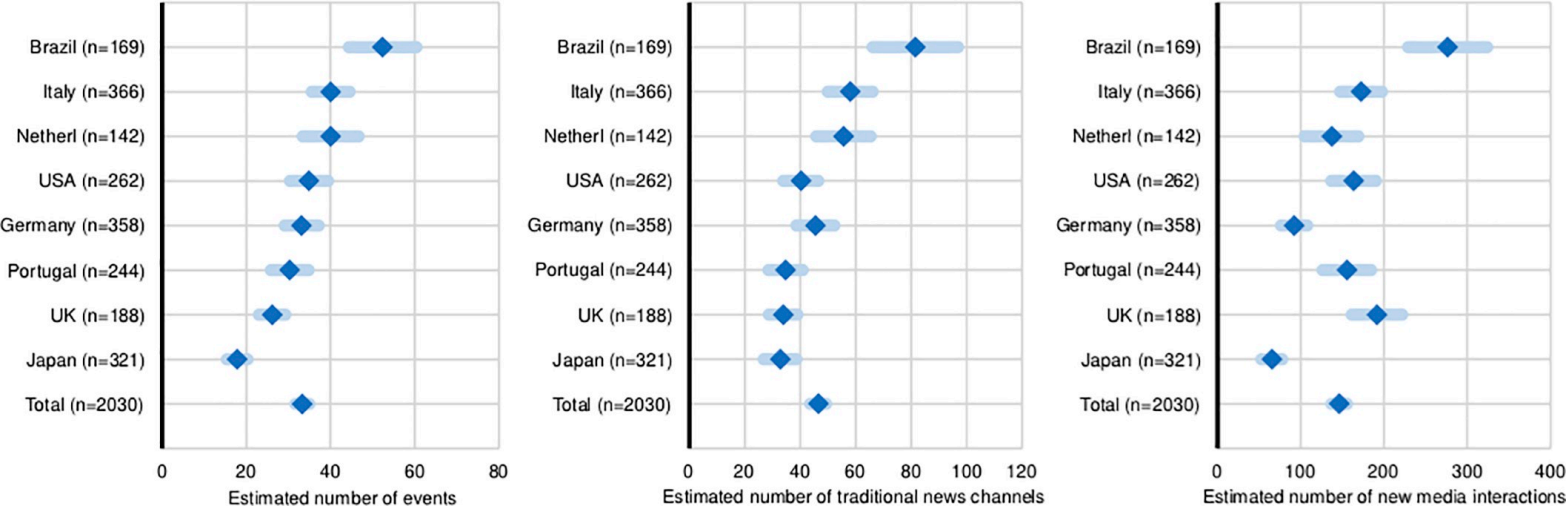
Frequency of public communication activity compared across countries. Estimated average number of public events, traditional news media, and new media channels by research institutes, in the twelve months prior to the study. (*N* = 2,030).

**Fig 2:**
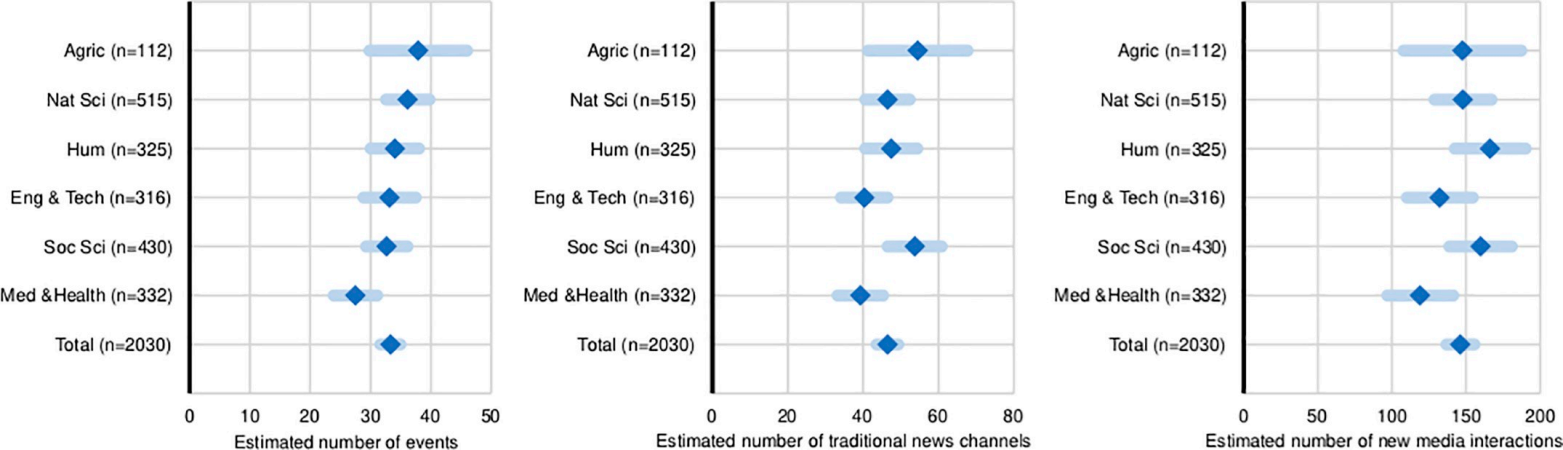
Frequency of public communication activity compared across sciences. Estimated average number of public events, traditional media, and social media channels by research institutes, in the twelve months prior to the study. (*N* = 2,030).

**Fig 3:**
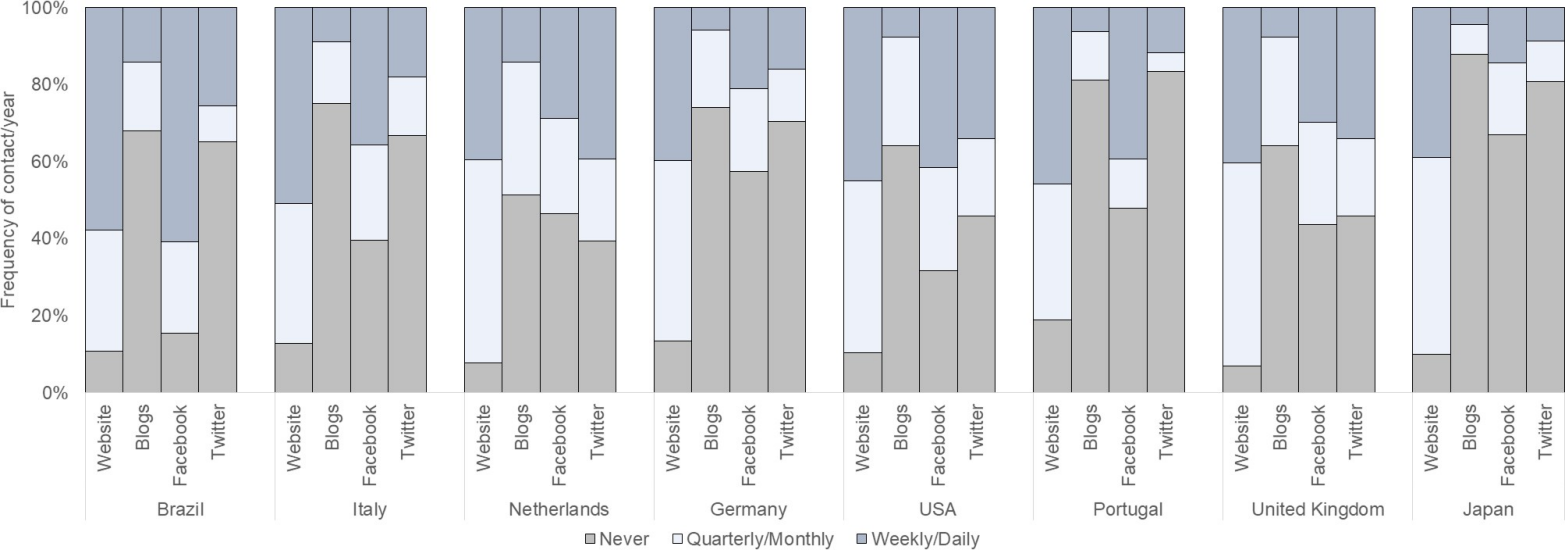
Frequency of new media channels used by country. Institutes were asked how frequently they used each online mean, on a 5-point scale form ‘never’ to ‘daily’.

**Fig 4:**
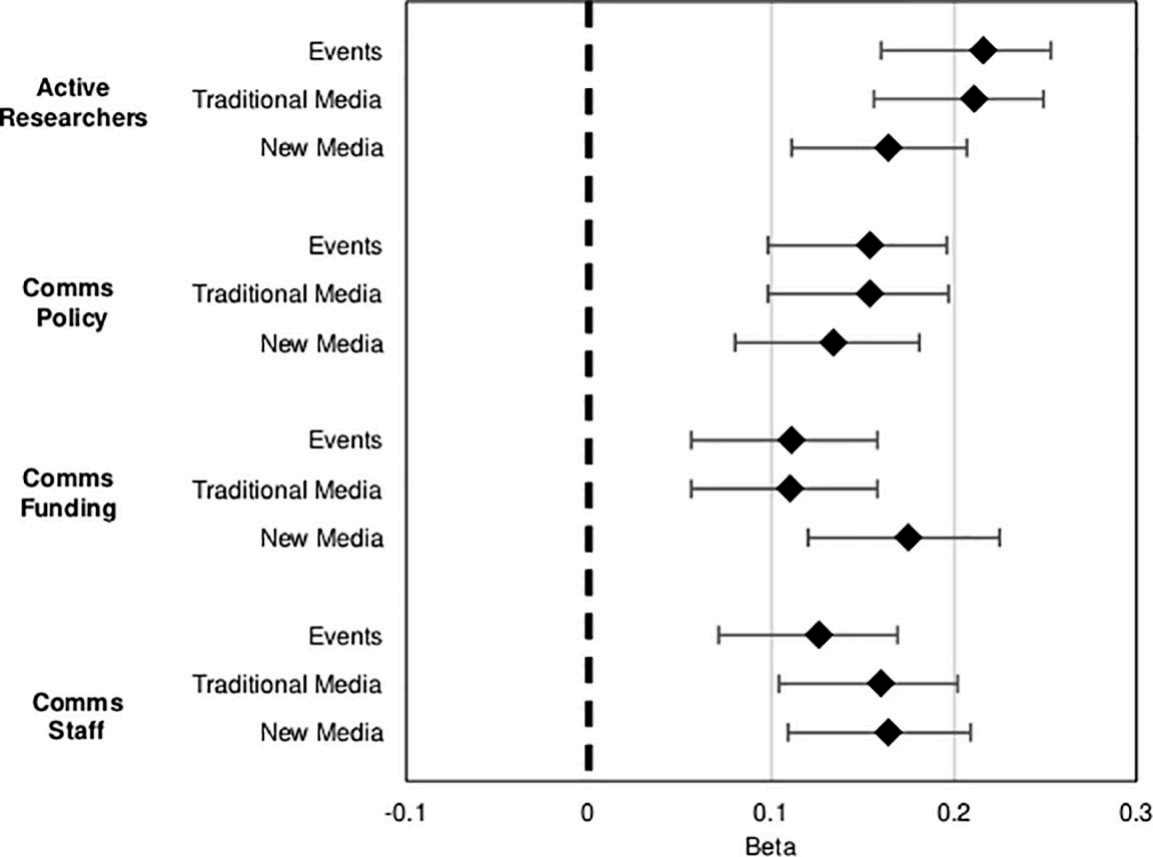
Forest plot explaining variation for level of public communication (dependent variables ‘public events’, ‘traditional news media’ and ‘new media’) regressed on context C and D variables; D variables are represented. Diamonds represent the standardised betas and the whiskers the 95% CIs (confidence intervals) (see S7–S9 Tables for representation of all variables in the models).

To address our goals, we asked institutes about the type and frequency of public communication activities they had organised or in which they had participated during the previous year, and how these were resourced. Public communication activities are any event or media activity to any non-peer public. To compare communication activities, we estimated one-way ANOVA models by country and research area (Figs 1–3). For these analyses, we used indices of number of public events, traditional news media, and new media channels. For the subsequent analyses of drivers of activity, we use continuous indices of intensity (see factor scores described in the Methods section) (Fig 4).

### Emerging public communication at the level of research institutes

The data show that most research institutes across the surveyed countries and scientific areas engage in a variety of public events and traditional media channels, and to a lesser extent in new media (Figs 1–3). As a benchmark, the median institute reports 21 public events (M = 33, SD = 0.9) and 25 media interactions (M = 46, SD = 1.5) per year respectively (only around 7% of the institutes did not report events and media contacts), and the median institute reports 52 online interactions per year (M = 146, SD = 4.8) (S3 Table). However, online interactions occur mostly through institutional websites (73% reported updating these at least monthly), with a large proportion of institutes reporting never using Facebook or Twitter (46% and 60% respectively) (Fig 3). We also find that overall, institutes more active in one type of means of public communication are also more active in the others (shown by the strong positive correlations between the indices (S10 Table). Yet, high activity is concentrated in a small subset of institutes; 30% of all institutes reported activities above the average frequency, in all means of communications.

Institutes also reported on their commitment to public communication. Half have adopted communication policies of some kind or the other, four in ten employ communications staff and half rely on central communications or PR offices to disseminate their news; the average institute spends around 3% of its annual research budget on public communications. This seems to indicate, that public communication is not yet fully institutional and taken-for-granted among research institutes; we find however evidence of a growing commitment over the past 5 years: respondents said public communication of science had increased in most institutes (61%) and started in another third; and expectations are for continuous growth with about half expecting to dedicate more resources in the coming years, and 90% expecting their research staff to engage in public communication.

### Differences across countries and areas of research

Figs 1 and 2 show public communication activity compared across countries and sciences. We find significant differences in levels of activity across countries and sciences, as determined by one-way between country and sciences ANOVAs (S6 Table), but these differences are overall small. Analyses using the Bonferroni post-hoc significance testing show statistically significant differences only among some of the countries and sciences. For all three dependent variables, the reported number of public events is significantly higher in Brazil (M = 52, SD = 28) than elsewhere (p<0.001), and significantly lower in Japan (M = 17, SD = 22) than elsewhere (p<0.001). There is no statistically significant difference in public event making among Italy (M = 40, SD = 43), Germany (M = 33, SD = 38), Portugal (M = 30, SD = 34), the Netherlands (N = 40, SD = 40), and the USA (M = 34, SD = 37) (p >.05), but activities are lower among UK institutes (M = 26, SD = 21) than in Italy, the Netherlands, and Brazil. Traditional media channels are used more frequently in Italy (M = 58, SD = 78) and Brazil (M = 81, SD = 101) than elsewhere. The differences in activity between Germany, Portugal, the Netherlands, the UK and the USA are not significant (p < .05). The use of new media is lower in Germany (M = 92, SD = 150) and Japan (M = 65, SD = 114) compared to the other countries (for example, the UK reports M = 192, SD = 209, and Brazil reports M = 276, SD = 312); though the Netherlands (M = 137, SD = 194) is not different from Germany (both showing lower activity). All other comparisons were insignificant (p < .05).

Fig 2 shows also small differences among the various sciences for public event making and traditional media, and no differences in the use of social media. Substantive differences are found between the medical sciences (M = 28, SD = 33) that engage less in public events and traditional media channels than agricultural sciences (M = 37, SD = 43) and the natural sciences (M = 36, SD = 42). All other comparisons are statistically insignificant (p>.05). These results confirm our expectations that there is variation in the activity of research institutes across countries and different scientific contexts, in the means they use.

### Drivers of institutional public communication activity

To examine what factors might predict the level of public communication activity in events, traditional channels, and new media, we run hierarchical multiple regressions, controlling for all other variables (**S7–S9** Tables).

Models 1 show that organisational context variables account for 13% of the variance. Institute size and research budget have a positive effect on all three main variables. These effects are kept significant in Models 2, controlling for all other conditions, further confirming the importance of these variables. Yet, it is interesting to note that size exerts a higher influence on event making (B = .127, p < .001) and traditional news media (B = .124, p < .001) than it does in social media (B = 0.06, p = 0.027). Larger institutes with larger research budgets tend to organise more public events and have a higher presence in traditional media. Country and research area also make important contributions, as we expected, and the effects of these variables remain significant in Models 2, further confirming the importance of country and disciplinary cultures for public communication of research institutes together with the size of the institute and the funding available for research.

D variables explain an additional 13–14% of the variance, for all three dependent variables (Models 2), when all other conditions are controlled (Fig 4). Models 2 thus explain the intensity of public communication above and beyond the variables in Models 1, and the increase in the R^2^ is statistically significant, and all commitment variables show positive associations with the level of activity. For example, Fig 4 shows that ‘active researchers’ explain a higher percentage of the variation in public events (B = .220, p < .001) and in traditional media (B = .210, p < .001) than the budget for communications (B = .011, p < .001; while the budget for communications (B = .180, p < .001) and communications staff (B = .160, p < .001) show stronger effects on the level of activity in social media than do the other commitment indicators (S7–S9 Tables). These findings support our model according to which both context C and disposition D factors contribute independently and jointly to the level of public communication of research institutes.

## Discussion

In this international study, we examined the public communication activities of research institutes across eight countries using random probability sampling stratified by research areas. We built on our exploratory research [1] to investigate factors that predict levels of activity in different formats of public communication, and report here for the first-time comparative evidence of activities across formats. We made three main observations.

Our first observation is that public communication remains far from being fully instituted and taken-for-granted across research institutes in universities and large research organisations. However, we found evidence for increasing capacity building and commitment of resources. Overall, traditional news media are the most used formats of communication by research institutes, and social media networks play only a marginal role. These results confirm our preliminary findings [1] and corroborate findings of another study in German universities [13], which show higher engagement in traditional news channels and only a small percentage of larger organisations engaging in social media. The large number of smaller institutes we survey here would be a structural reason for the reluctance to invest in new media. In our study, however, size is not among the most important determinants for the use of social media, but rather the resources that institutes have available. This is a surprise for us given that fast, low effort, and wide reach of new media platforms, which could play in favour of smaller institutes with less resources. Yet, the findings suggest that in the countries surveyed, smaller institutes that are more committed to public communication can have a higher profile in social media. We note, however, that concerning digital communication, while the website is the most used channel by almost all the surveyed countries, social media channels are popular among Brazilian institutes, particularly Facebook with 34% using it daily. This corroborates findings from other sources which confirm this trend in social media use in Brazil [36] [37].It is also possible that, to a certain extent, the communication practice of the institutions reflects the preferences of the demand side. In all the surveyed countries, most people still obtain their news about science from the traditional news media [38] [39]. Social media play almost no role as a source of information about science and technology among general audiences (e.g. 65% of European citizens get their information from television and 10% from social media or blogs) [38]. Thus, a scientific institution with limited resources acts rationally if it does not use social media, which is largely free of science audience. Also, we cannot rule out that institutes attach lower value to such channels for reaching their intended communication goals and audiences. Social media have been hyped to foster science communication [40], yet real evidence for this is limited or contradictory. For instance, social media have a poor reputation in Germany, especially among the more educated population, including scientists [41]. They are regarded as the playground of conspiracy theorists, as well as the place where small communities of opinion constantly confirm their own prejudices in largely closed bubbles. Moreover, many scientists consider it neither possible nor useful to convey the complexity of their research in short news items (tweets) [41], and the use of social media to communicate with the broad public has been found low among scientists, regardless of the discipline [26] [25].

Also, it was perhaps surprising for us to find such a high predominance of face-to-face events. However, these figures on public events could suggest public engagement being ‘event-focused’ at the level of the institutes. And, if we consider institutional events and new institutional media together, this may indicate that institutes are more in control of their public science communication and less dependent from the traditional gate keepers in the news media, which may be more in evidence at the central communications level Yet, this claim needs further investigation.

A second observation concerns variation: public communication activities differ across countries and sciences, yet, the small differences found were to a certain extent, unexpected for us. This confirms that regarding institutional communication, factors other than country and disciplinary cultures need to be considered, further supporting the validity of our model. Nevertheless, the differences found allow us to say that country and scientific area determine in part the level of communication activity in which a research institute engages. This is not entirely unexpected as countries have different national histories of science and education [42] [43] and commit to societal engagement of science in different ways through, for instance, policy imperatives, public debates, government directives, or promote some sciences before others (different public visibility of disciplines). For instance, the somewhat higher level of outreach activity in the agricultural sciences may go back to the tradition of USA land-grant universities and their extension services [44], which expanded internationally to many countries [45] [46]. It might also reflect the many controversies over the years on agriculture related topics (climate change, biofuels, or genetically modified crops). The ready presence of interested stakeholders (e.g. farmers) and the applied nature of the research might also be relevant. These factors might well have boosted communications structures in these fields. In contrast, the medical and health sciences may not have faced such stimuli towards public engagement, which while not entirely clear for us, may find some explanation in their communication focusing around the individual doctor-patient relationship [47]. But the fact that the medical sciences may be more susceptible to politicisation and driven by external funding for research [48] [49], may also explain part of this phenomenon.

Also, the similar level of communication activity across the countries surveyed allows us to say that differences are more in evidence if we compare global regions rather than individual countries. Europe and North America seem to perform similarly, compared to Brazil (South America) and Japan (Asia). Studies of scientists, although not directly comparable, have also pointed to this issue. For example, a five-country survey of medical scientists showed slightly higher media interactions among Western scientists (USA, France, Germany and UK) [28] than among scientists in Japan, and Entradas and Bauer (2019) [25] found lower interactions with the media and the public more broadly among astronomers in Asia compared to other global regions. This could suggest that science communication has continental features, but this needs further investigation as our data are limited to a small number of countries. Brazil generally shows higher levels of activity; this could be related to the fact that Brazilian research institutes in our sample are on average larger than elsewhere, and larger units generally communicate more.

Thirdly, we show that variation in communication is associated with institutional commitment to public communication such as having a policy in place, professional communications staff and available funding. We cannot however conclude whether more activity is a result of more resources, or if it is because institutions see communication as an important activity and duty, and thus dedicate more resources to it. For example, there could be a policy and staff because an institution is very active (instead of the other way around).But, overall, this seems to suggest that research institutes seeking to increase their public visibility employ professional communications staff to build media relations and to build a social media profile, while research scientists remain the main protagonists of public events.

Our observations urge us to think about public communication of science not as a country or discipline feature, but a profile arising from an ordered combination of context (C) and disposition (D) factors. For example, having professional staff will not lead to higher activity, but if there are no staff, an institute is unlikely to become highly active. These factors determine in combination the level of different types of communication activities of research institutes showed here. This might be particularly visible in market-oriented country universities such as the USA and UK, while in Italy and Germany the institutional ethos is stronger and communication is seen as a public good.

## Conclusions

Our data point to a growing international phenomenon and a potential change in the culture of academic institutions to open up their research to unspecific publics at the level of research institutes. Such opening up is well documented at policy level, at the level of universities, or at the level of individual researchers building a public profile. Here we document this phenomenon also at the level of research institutes. This change has been embraced across countries and disciplines, with varying intensity. Our results support our expectations that organisational context and disposition factors explain this variance, and we provide baseline measurements for institutional public communication. From this research new questions emerge that call for further study. For example, what are the consequences of this resource mobilisation and what are the implications of this professionalisation for the conduct of science itself and for the public conversation about science? Does this mean that resources hitherto earmarked for research are increasingly rerouted into communication, and scientists are losing control over their story telling? Is this professionalization of public communication consistent with a medialisation of science and the strategic over-adaptation of science towards publicity and reputation [50]? Are professionalised staff a gain to science communication? Is this development increasing the autonomy and values of science or becoming a prisoner of a logic of competing for public visibility [51]? Most people consider more communication a better thing. However, we might have to consider here as elsewhere the unintended consequence of good intentions. Also, at the level of institutes, public communication of research might turn into a marketing exercise and detach itself from the original aims of public engagement, which are to contribute to public debates. An ‘arms race’ for public visibility between research institutes could bias the research system towards non-research activities and thus risk undermining core research activities, not least for the smaller players. Future research must investigate the implications of this professionalisation for science communication and the narratives of this public communication that emerges from research institutes, as well as the values that support this effort.

### Limitations

This study reports the largest and most systematic survey of research institutes’ practices of public communication of science so far, and being one of the larger surveys of science communication in scope and comparability marks an important contribution to the empirical literature on public engagement of science. But no study answers all possible questions, nor all the questions beyond reasonable doubt. There is a need for further investigation in other regions where there were a limited number of countries such as Asia and South America, but it should also extent to other regions including Africa to better understand the variation of activity across global regions. Also, our models explain only a part of the variance of public communication activity. Other factors should be brought into consideration to improve explanatory power.

## Supporting information

S1 Table Sampling frames and procedures employed in each country. (DOCX)

S2 Table Number of institutions contacted (N), number of institutions that responded (N) by country and areas of research, unweighted (RR) and weighted response rates (WRR). (DOCX)

S3 Table Descriptive for indices from sum of activities. (DOCX)

S4 Table (a) (b), and (c) Confirmatory factor analysis loadings for variables public events, traditional channels and new media.
(DOCX)

S5 Table Descriptive statistics for main variables. (DOCX)

S6 Table Analysis of variance by country and area of research. Abreviations: Sum of squares (SS); df (degrees of freedom); MS (Mean Square); F statistic (F) and (p (significance value), and Eta (strength of the relationship).
(DOCX)

S7 Table Hierarchical regression analysis for public communication activity in public events showing the two-step analysis. (DOCX)

S8 Table Hierarchical regression analysis for public communication activity in traditional media channels showing the two-step analysis. (DOCX)

S9 Table Hierarchical regression analysis for public communication activity in social media channels showing the two-step analysis. (DOCX)

S10 Table Correlations between main variables. (DOCX)

S1 Text (DOCX)

S1 File (DOCX)

## References

[1] Entradas M, Bauer MW. Mobilisation for public engagement: Benchmarking the practices of research institutes. Public Underst Sci. 2017; 26(7):771–88. 10.1177/0963662516633834 26951156

[2] Leshner A. Public engagement with science. Science. 2003; 299(5609):977 10.1126/science.299.5609.977 12586907

[3] Marcinkowski F, Kohring M, Fürst S, Friedrichsmeier A. Organizational Influence on Scientists’ Efforts to Go Public: An Empirical Investigation. Sci Commun. 2014; 36(1):56–80.

[4] McAllister SM. How the world’s top universities provide dialogic forums for marginalized voices. Public Relat Rev. 2012;38(2):319–27.

[5] Laredo P. Revisiting the third mission of universities: Toward a renewed categorization of university activities? High Educ Policy. 2007;20(4):441–56.

[6] Commission European. Public Engagement in Science—Report of the Science and Society Session, Portuguese Presidency Conference, The Future of Science and Technology in Europe. 2007 https://ec.europa.eu/research/swafs/pdf/pub_other/public-engagement-081002_en.pdf

[7] Options for Strengthening Responsible Research and Innovation. http://europa.eu

[8] Weingart P, Pansegrau P. Reputation in science and prominence in the media: the Goldhagen debate. Public Underst Sci. 1999; 8(1):1–16. 10.1088/0963-6625/8/1/001

[9] Rödder S. Science and the mass media -’medialization’ as a new perspective on an intricate relationship. Sociol Compass. 2011 9; 5(9):834–45.

[10] Rowe D, Brass K. The uses of academic knowledge: The university in the media. Media, Cult Soc. 2008;30(5):677–98.

[11] Peters HP. Scientific Sources and the Mass Media: Forms and Consequences of Medialization. In: Rödder S., Franzen M. WP (eds). The Sciences’ Media Connection–Public Communication and its Repercussions. 2012 p. 217–39.

[12] Rowe D, Brass K. “We take academic freedom quite seriously”: How university media offices manage academic public communication. Int J Media Cult Polit. 2011;7(1):3–20.

[13] Metag J, Schäfer MS. Hochschulen zwischen Social Media-Spezialisten und Online-Verweigerern. Eine Analyse der Online-Kommunikation promotionsberechtigter Hochschulen in Deutschland, Österreich und der Schweiz. Stud Commun Media. 2017 6 28;6(2):160–95.

[14] Neresini F, Bucchi M. Which indicators for the new public engagement activities? An exploratory study of European research institutions. Public Underst Sci. 2011;20(1):64–79. 10.1177/0963662510388363

[15] Entradas M, Bauer MW. Kommunikationsfunktionen im Mehrebenensystem Hochschule In: Forschungsfeld Hochschulkommunikation. Wiesbaden: Springer Fachmedien Wiesbaden; 2018 p. 97–122.

[16] Mejlgaard N, Bloch C, Madsen EB. Responsible research and innovation in Europe: A cross-country comparative analysis. Sci Public Policy. 2018;46(2):198–209.

[17] Commission European. Europeans, Science and Technology. Brussels; 2005.

[18] OECD Science, Technology and Industry Outlook 2014 OECD Publishing.

[19] National Academy of Sciences, Engineering and M. Communicating science effectively: A research agenda. National Academies Press; 2017. 28406600

[20] Fischhoff B. The sciences of science communication. Proc Natl Acad Sci. 2013;110. 10.1073/pnas.1213273110 PMC3752164 23942125

[21] OECD Frascati Manual. Proposed Standard Practise for Surveys on Research and Experimental Development. 2015.

[22] Bauer MW, Jensen P. The mobilization of scientists for public engagement. Public Underst Sci. 2011;20(1):3–11.

[23] Jensen P. A statistical picture of popularization activities and their evolutions in France. Public Underst Sci. 2011;20(1):26–36.

[24] Besley JC, Dudo A, Yuan S. Scientists’ views about communication objectives. Public Underst Sci. 2018;27(6):708–30. 10.1177/0963662517728478 28841818

[25] Entradas M, Bauer MW. Bustling public communication by astronomers around the world driven by personal and contextual factors. Nat Astron. 2019;3(2):183–7.

[26] Entradas M, Marcelino J, Bauer MW, Lewenstein B. Public communication by climate scientists: what, with whom and why? Clim Change. 2019;154(1–2):69–85.

[27] Bentley P, Kyvik S. Academic staff and public communication: a survey of popular science publishing across 13 countries. Public Underst Sci. 2011;20(1):48–63.

[28] Peters HP, Brossard D, De Cheveigné S, Dunwoody S, Kallfass M, Miller S, et al Science communication: Interactions with the mass media. Science. 2008;321(5886):204–5. 10.1126/science.1157780 18625578

[29] Entradas M et al The late bloom of (modern) science communication in Portugal In: Gascoigne T, editor. The emergence of modern science communication. ANU Press.

[30] Lewin K. Principles of Topological Psychology. McGraw-Hill, editor. New York; 1936.

[31] Sekaran U. Methodological and Theoretical Issues and Advancements in Cross-Cultural Research. J Int Bus Stud. 1983;14(2):61–73.

[32] Dillman DA. Mail and telephone surveys: the total design method New York: John Wiley & Sons; 1978.

[33] O’Muircheartaigh C, Eckman S, Smith S. Statistical Design and Estimation for the National Social Life, Health, and Aging Project. Journals Gerontol Ser B Psychol Sci Soc Sci. 2009;64B(Supplement 1):i12–9. 10.1093/geronb/gbp045 PMC2763522 19567827

[34] Shih TH, Xitao F. Comparing response rates from web and mail surveys: A meta-analysis. Field methods. 2008;20(3):249–71.

[35] Sheehan KB. E-mail Survey Response Rates: A Review. J Comput Commun. 2006;6(2).

[36] Centro de Gestão e Estudos Estratégicos. Percepção pública da C&T no Brasil– 2019. Resumo executivo Brasília, DF: 2019 24p. Disponível em: https://www.cgee.org.br/documents/10195/734063/CGEE_resumoexecutivo_Percepcao_pub_CT.pdf

[37] GlobalWebIndex (2019). Social media trends in 2019.https://www.globalwebindex.com/reports/social-2019

[38] Eurobarometer Special 401—Responsible Research and Innovation (RRI), Science and Technology. 2013 10.1142/S2339547813500027

[39] National Science Board. Science and Technology Indicators. Washington, DC; 2016.

[40] Hargittai E, Füchslin T, Schäfer MS. How Do Young Adults Engage With Science and Research on Social Media? Some Preliminary Findings and an Agenda for Future Research. Soc Media + Soc. 2018.

[41] Rümmele K. Öffentliche Wissenschaft und Neue Medien: Die Rolle der Web 2.0-Kultur in der Wissenschaftsvermittlung Robertson-von Trotha CY& JMM, editor. Karlsruhe: KIT Scientific Publishing; 2012 157–168.

[42] Huff T. The rise of early modern science: Islam, China, and the West. 2017.

[43] Cunningham Peter, Taylor R. Beyond the lecture hall: universities and community engagement from the middle ages to the present day. Hist Educ. 2008;41(2).

[44] Mcdowell GR. Engaged Universities: Lessons from the Land-Grant Universities and Extension. Ann Am Acad Pol Soc Sci. 2003; 585(1):31–50.

[45] Weerts David J., Sandmann Lorilee R. Building a Two-Way Street: Challenges and Opportunities for Community Engagement at Research Universities. Rev High Educ. 2008;32(1):73–106.

[46] Neset T-S, Asplund T, Käyhkö J, Juhola S. Making sense of maladaptation: Nordic agriculture stakeholders’ perspectives. Clim Change. 2019;153(1–2):107–21.

[47] Domecq JP, Prutsky G, Elraiyah T, Wang Z, Nabhan M, Shippee N, et al Patient engagement in research: a systematic review. BMC Health Serv Res. 2014;14(1):89. 24568690 10.1186/1472-6963-14-89 PMC3938901

[48] Katz Y, Matter U. On the Biomedical Elite: Inequality and Stasis in Scientific Knowledge Production.SSRN. Berkman Klein Center for Internet & Society; 2017.

[49] Murray DL, Morris D, Lavoie C, Leavitt PR, MacIsaac H, Masson MEJ, et al Bias in research grant evaluation has dire consequences for small universities. PLoS One. 2016;11(6). 10.1371/journal.pone.0155876 PMC4892638 27258385

[50] Weingart P, Maasen S. Elite Through Rankings–The Emergence Of the Enterprising University. In Springer, Dordrecht; 2007; 75–99.

[51] Weingart P, Engels A, Pansegrau P. Risks of communication: discourses on climate change in science, politics, and the mass media. Public Underst Sci. 2000;9(3):261–83.

