# Correction: Public communication by research institutes compared across countries and sciences: Building capacity for engagement or competing for visibility?

**DOI:** 10.1371/journal.pone.0242950

**Published:** 2020-11-20

**Authors:** Marta Entradas, Martin W. Bauer, Colm O'Muircheartaigh, Frank Marcinkowski, Asako Okamura, Giuseppe Pellegrini, John Besley, Luisa Massarani, Pedro Russo, Anthony Dudo, Barbara Saracino, Carla Silva, Kei Kano, Luis Amorim, Massimiano Bucchi, Ahmet Suerdem, Tatsuo Oyama, Yuh-Yuh Li

The affiliations for the 11th and 15th authors are incorrect. The correct affiliations are as follows: Barbara Saracino^11^, Massimiano Bucchi^13^

**11** Department of Political and Social Sciences, University of Bologna, Bologna, Italy, **13** Department of Sociology, Università di Trento, Trento, Italy

Information is missing in the captions for Figs 1 and 2. Please see the correct Fig 1 and Fig 2 captions here.

**Fig 1:**
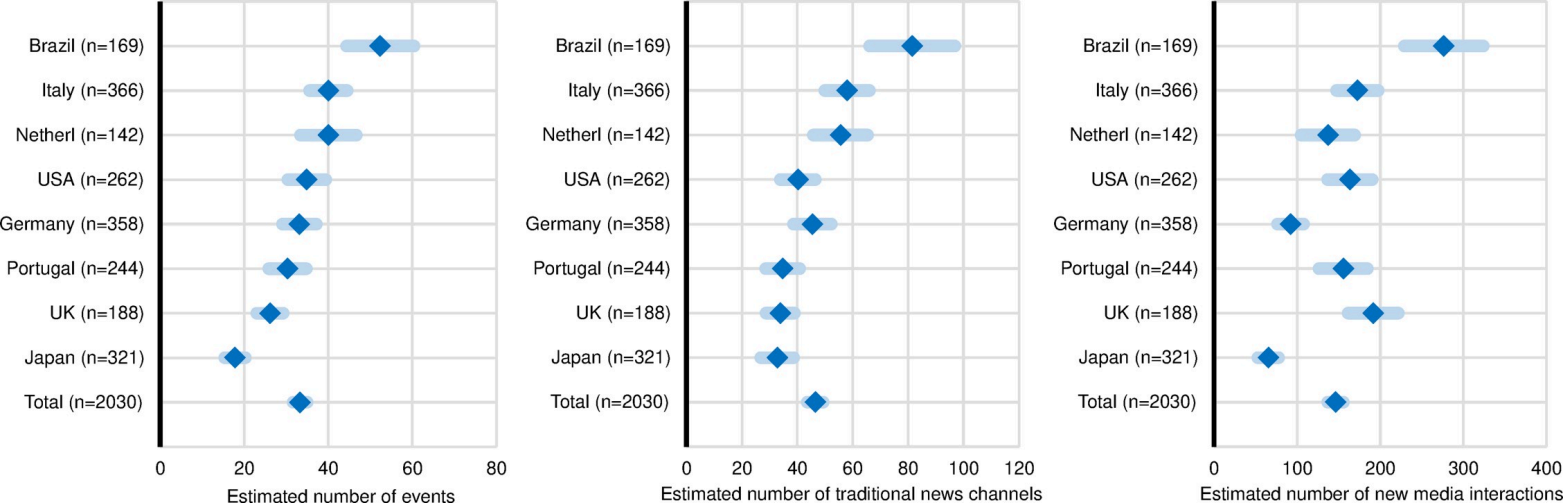
Frequency of public communication activity compared across countries. Estimated average number of public events, traditional news media, and new media channels by research institutes, in the twelve months prior to the study. Diamonds represent the means and the light shaded bars the 95% CIs (confidence intervals) (*N* = 2,030).

**Fig 2:**
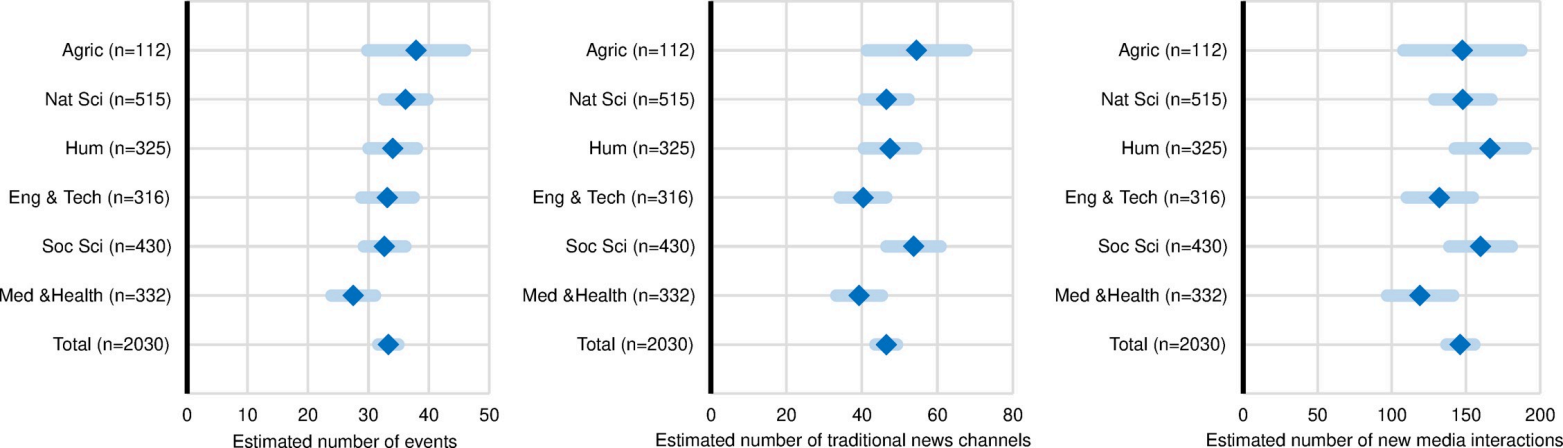
Frequency of public communication activity compared across sciences. Estimated average number of public events, traditional media, and social media channels by research institutes, in the twelve months prior to the study. Diamonds represent the means and the light shaded bars the 95% CIs (confidence intervals) (*N* = 2,030).
