# Supplementary material for: Public communication by research institutes compared across countries and sciences: Building capacity for engagement or competing for visibility?
Source: PLoS One. 2020 Jul 8;15(7):e0235191. doi: 10.1371/journal.pone.0235191 (PMC7343166; doi:10.1371/journal.pone.0235191)
Supplement: S1 Table — (DOCX) [file pone.0235191.s001.docx]

**S1 Table.** Sampling frames and procedures employed in each country.

| **Country** | **Sampling  frame (N)** | **Target  sample (N)** | **Sampling procedure** |
| --- | --- | --- | --- |
| Brazil | N=945 | N=945 | To build the Brazilian target sample, we identified all public Brazilian research institutes linked to Ministries (N= 46) (non-university institutes) and used the census (2015) provided by the INEP (Instituto Nacional de Estudos e Pesquisas Educacionais Anísio Teixeira) for a complete picture of the brasilian universities (N= 2,368). We selected N=50 best universities using a combination of the lists provided by The Times Higher Education (2017) and the Ranking of the Brazilian newspaper Folha de São Paulo (2017). We listed all research institutes within the selected universities (N=899) and achieved a sampling frame of N=945. We contacted all institutions. |
|  |  |  |  |
| Portugal | N=384 | N=384 | We identified all research institutes in Portugal from two official lists: the list of research institutes funded by FCT (Fundação para a Ciência e Tecnologia, 2014) and Portuguese Higher Education Institutions list provided by the DGEES, Direção-Geral de Estatísticas do Ensino Superior, 2014). The final sampling the frame resulted in N=384 research institutes. This represents at least 80% of the population. We contacted all institutions. |
| Germany | N=1,900 | N=1,358 | We identified N=193 state run Research Universities & Universities of Applied Science, from an official list provided by the Association of University Presidents (HRK) (2017) (Liste aller Mitgliederhochschulen der Hochschulrektorenkonferenz); this covers more than 90% of all academic staff in the German Higher education system. We randomly assigned one of the six research fields (OECD classification) to each university, so that each research field was covered by approx. 30 organisations. We then listed all research institutes of the respective field for each of the universities. This resulted in at least 10 institutes per University and a total sample of about 1.900 institutes (around 300 per OECD-Field), from which we draw a random sample for each field. |
|  |  |  |  |
| Italy | N=1,120 | N=1,120 | To build the Italian sample, we collected lists of all Italian Universities and Research Institutes from the Italian Ministry of Education and Research (MIUR) database (2017). We then made a complete list of all institutes within these universities, which corresponded to the Universities Departments and Italian Research Institutes. We classified each unit based on the OECD area of research, and contacted the entire population (N=1,120). |
|  |  |  |  |
| Japan | N=1,134 | N=1,134 | To build the Japanese frame, we used a list from the “National Institute of Science and Technology Policy” (NISTEP) (2016), which covers all R&D conducted at universities and corporations in Japan (N=787); we selected the top 66 universities, based on KAKEN database (this covers around 80% of KAKEN grant in 2016). KAKEN is the grants-in-aid for scientific research, which is operated by Japanese Society for the Promotion of Science (JSPS) and the biggest competitive funding agency in Japan. For each of the 66 universities, we listed all research institutes within each organization (N=1,134). All institutes were contacted. |
|  |  |  |  |
| Netherlands | N=821 | N=821 | To build the NL frame, we used a list provided by Narcis (2016) of all universities (N=14), and Para-university organizations: the Netherlands Organization for Scientific Research (NWO) (N=8) and the Royal Netherlands Academy of Arts and Sciences (KNAW) (N=16). We mapped all institutes within these organisations using information from their websites, and classified each of them according to OECD field (N=821). As the total number of Dutch research institutes was smaller than the 200 per group, we contacted the whole population. |
| United  Kingdom | N=1,957 | N=1,046 | For the UK sampling frame, we used the list (census) of UK universities provided by the Research Excellence Framework (2015) (N=150). The REF is the new system for assessing the quality of research in UK higher education institutions. Universities were ranked into HH (high research output; high research impact), HL, LH; and LL, and a N=50 covering all ranks was randomly selected; all universities from the Russel Group were added. All research institutes within the selected universities were mapped using information from universities' websites, and classified in OECD field (N=1,957). N=200 from each sector was randomly selected, plus some hand pick of institutes in the agricultural sciences outside universities (N=10), which were underrepresented, resulting in N=1,046. |
| United States of America | N=10, 308 | N=1,366 | To build the American sampling frame, the mapped all research institutes within the “Doctoral Universities: Very High Research Activity” classified by the Carnegie  Foundation (2017); this resulted in (N=10,308) research units. Each was classified into OECD field. For each university, a random number generator was used to select two units for each category (i.e., 12 units per university or 230 per OECD group). In cases where a university did not have a relevant centre or institute, units were selected from other universities at random. It was not possible to identify enough agricultural science units such that the final contact list included 1,366 potential respondents. |
