# Supplementary material for: Public communication by research institutes compared across countries and sciences: Building capacity for engagement or competing for visibility?
Source: PLoS One. 2020 Jul 8;15(7):e0235191. doi: 10.1371/journal.pone.0235191 (PMC7343166; doi:10.1371/journal.pone.0235191)
Supplement: S2 Table — (DOCX) [file pone.0235191.s002.docx]

|  | | **Institutions contacted  (*N)* (a)** | **Institutions responded  (*N*)** | **Response Rate**  **(RR) (%) (b)** | **Weighted RR (%) (c)** |
| --- | --- | --- | --- | --- | --- |
| **United Kingdom** | Nat Sci | 185 | 33 | 17.8 | 31.8 |
|  | Eng & Tech | 191 | 22 | 11.5 | 14.8 |
|  | Med & Health | 187 | 32 | 17.1 | 26.2 |
|  | Agric Sci | 26 | 11 | 42.3 | 25.4 |
|  | Soc Sci | 191 | 50 | 26.2 | 53.3 |
|  | Hum | 187 | 40 | 21.4 | 30.3 |
|  | **Total** | **967** | **188** | **19.4** | **31.0** |
| **Germany** | Nat Sci | 357 | 122 | 34.2 | 31.5 |
|  | Eng & Tech | 258 | 59 | 22.9 | 23.5 |
|  | Med & Health | 217 | 42 | 19.4 | 16.0 |
|  | Agric Sci | 120 | 21 | 17.5 | 18.0 |
|  | Soc Sci | 213 | 61 | 28.6 | 26.2 |
|  | Hum | 191 | 53 | 27.7 | 26.8 |
|  | **Total** | **1356** | **358** | **26.4** | **23.7** |
| **United States** | Nat Sci | 227 | 63 | 27.8 | 28.1 |
|  | Eng & Tech | 221 | 26 | 11.8 | 11.2 |
|  | Med & Health | 219 | 42 | 19.2 | 21.2 |
|  | Agric Sci | 221 | 29 | 13.1 | 19.2 |
|  | Soc Sci | 223 | 54 | 24.2 | 31.5 |
|  | Hum | 220 | 48 | 21.8 | 30.2 |
|  | **Total** | **1331** | **262** | **19.7** | **23.6** |
| **Netherlands** | Nat Sci | 116 | 30 | 25.9 |  |
|  | Eng & Tech | 105 | 16 | 15.2 |  |
|  | Med & Health | 164 | 25 | 15.2 |  |
|  | Agric Sci | 12 | 2 | 16.7 |  |
|  | Soc Sci | 302 | 44 | 14.6 |  |
|  | Hum | 122 | 25 | 20.5 |  |
|  | **Total** | **821** | **142** | **17.3** |  |
| **Japan** | Nat Sci | 245 | 66 | 26.9 |  |
|  | Eng & Tech | 225 | 70 | 31.1 |  |
|  | Med & Health | 194 | 67 | 34.5 |  |
|  | Agric Sci | 42 | 16 | 38.1 |  |
|  | Soc Sci | 318 | 70 | 22.0 |  |
|  | Hum | 110 | 32 | 29.1 |  |
|  | **Total** | **1134** | **321** | **28.3** |  |
| **Italy** | Nat Sci | 247 | 98 | 39.7 |  |
|  | Eng & Tech | 188 | 70 | 37.2 |  |
|  | Med & Health | 227 | 64 | 28.2 |  |
|  | Agric Sci | 52 | 16 | 30.8 |  |
|  | Soc Sci | 252 | 63 | 25.0 |  |
|  | Hum | 154 | 55 | 35.7 |  |
|  | **Total** | **1120** | **366** | **32.7** |  |

|  |  | **Institutions contacted  (*N)* (a)** | **Institutions responded  (*N*)** | **Response Rate**  **(RR) (%) (b)** |  |
| --- | --- | --- | --- | --- | --- |
| **Brazil** | Nat Sci | 238 | 49 | 20.6 |  |
|  | Eng & Tech | 105 | 17 | 16.2 |  |
|  | Med & Health | 167 | 31 | 18.6 |  |
|  | Agric Sci | 61 | 9 | 14.8 |  |
|  | Soc Sci | 250 | 32 | 12.8 |  |
|  | Hum | 99 | 31 | 31.3 |  |
|  | **Total** | **920** | **169** | **18.4** |  |
| **Portugal** | Nat Sci | 94 | 54 | 57.4 |  |
|  | Eng & Tech | 67 | 36 | 53.7 |  |
|  | Med & Health | 45 | 29 | 64.4 |  |
|  | Agric Sci | 14 | 8 | 57.1 |  |
|  | Soc Sci | 98 | 56 | 57.1 |  |
|  | Hum | 66 | 41 | 62.1 |  |
|  | **Total** | **384** | 224 | **58.3** |  |
| **All countries** | Nat Sci | 1709 | 515 | 30.1 |  |
|  | Eng & Tech | 1360 | 316 | 23.2 |  |
|  | Med & Health | 1420 | 332 | 23.4 |  |
|  | Agric Sci | 548 | 112 | 20.4 |  |
|  | Soc Sci | 1847 | 430 | 23.3 |  |
|  | Hum | 1149 | 325 | 28.3 |  |
|  | **Total** | **8033** | **2030** | **25.3** |  |
| (a) This number has been adjusted to exclude non-eligible cases including non-RIs, extinct RIs, no valid email address found. | | | | | |

**S2 Table. Number of institutions contacted (N), number of institutions that responded (N) by country and areas of research, unweighted (RR) and weighted response rates (WRR).** For every country we present column the unweighted response rate; for countries where we undertook a nonresponse mitigation approach by subsampling nonrespondents and approaching the subsample again (UK, Germany, and USA), we present the weighted response rate (4), which uses those supplementary cases to represent nonrespondents as a whole. This is the best representation of the extent to which the sample mirrors the population.

(b) The unweighted response rate (RR) is essentially the ratio of the number of responses to the number of cases approached.

(c) The weighted response rate (WRR) takes the responses, each weighted by the inverse of its probability of inclusion as a proportion of the sum of the weights across the whole population. This is a better guide to the potential for response bias than the unweighted response rate.
