# Supplementary material for: Public communication by research institutes compared across countries and sciences: Building capacity for engagement or competing for visibility?
Source: PLoS One. 2020 Jul 8;15(7):e0235191. doi: 10.1371/journal.pone.0235191 (PMC7343166; doi:10.1371/journal.pone.0235191)
Supplement: S3 Table — (DOCX) [file pone.0235191.s003.docx]

**S3 Table.** Descriptives for indices from sum of activities.

|  | **Mean** | **Std. Error** | **Median** | **Bottom error** | **Top of error** | **Range** | **Cronbach’s** a |
| --- | --- | --- | --- | --- | --- | --- | --- |
| Public Events | 33.30 | 0.85 | 21 | 32.46 | 34.15 | 0-260 | 0.70 |
| Traditional media | 46.45 | 1.46 | 25 | 44.99 | 47.90 | 0-529 | 0.85 |
| New media | 146.20 | 4.78 | 52 | 141.42 | 150.98 | 0-1440 | 0.72 |
