# Supplementary material for: Public communication by research institutes compared across countries and sciences: Building capacity for engagement or competing for visibility?
Source: PLoS One. 2020 Jul 8;15(7):e0235191. doi: 10.1371/journal.pone.0235191 (PMC7343166; doi:10.1371/journal.pone.0235191)
Supplement: S4 Table — (a) (b), and (c) Confirmatory factor analysis loadings for variables public events, traditional channels and new media. (DOCX) [file pone.0235191.s004.docx]

**S4(a) (4b), and 4(c) Tables. Confirmatory factor analysis loadings for variables public events, traditional channels and new media.** Model fit statistics (Chi-square (χ2), degrees of freedom (df), Chi-square to df ratio, Comparative Fit Index (CFI), Root Mean Square Error of Approximation (RMSEA), Tucker-Lewis index (TLI), and Bayesian Information Criterion (BIC). (4c) Measurement invariance fit for country.

(4a)

| **Latent  variables** | **Standardized load** | **Std.Err** | **z-value** | **P(>\|z\|)** |
| --- | --- | --- | --- | --- |
| *Public events* |  |  |  |  |
| Public lectures | 0.434 | 0.016 | 27.913 | 0.000 |
| exhibitions | 0.329 | 0.013 | 24.478 | 0.000 |
| Open days | 0.355 | 0.014 | 25.027 | 0.000 |
| Sci fest | 0.310 | 0.013 | 24.754 | 0.000 |
| Sci_cafes | 0.324 | 0.014 | 23.109 | 0.000 |
| Talks scho | 0.383 | 0.015 | 24.746 | 0.000 |
| Citizen sci | 0.312 | 0.014 | 22.036 | 0.000 |
| *Trad channels* |  |  |  |  |
| Inter news | 0.482 | 0.014 | 33.674 | 0.000 |
| Inter radio | 0.447 | 0.014 | 31.487 | 0.000 |
| Inter TV | 0.430 | 0.014 | 31.234 | 0.000 |
| Press conf | 0.266 | 0.013 | 21.155 | 0.000 |
| Press releases | 0.450 | 0.016 | 27.981 | 0.000 |
| Articles magz | 0.460 | 0.014 | 32.658 | 0.000 |
| Popular books | 0.284 | 0.012 | 22.927 | 0.000 |
| *New media* |  |  |  |  |
| Twitter | 0.310 | 0.011 | 27.835 | 0.000 |
| Blogs | 0.248 | 0.011 | 22.927 | 0.000 |
| Facebook | 0.301 | 0.012 | 26.034 | 0.000 |
| YouTube | 0.318 | 0.011 | 28.807 | 0.000 |
| Podcasts | 0.152 | 0.009 | 17.563 | 0.000 |

| χ2 | **df** | **CFI** | **RMSEA** | **TLI** | **BIC** |
| --- | --- | --- | --- | --- | --- |
| 627.54 | 142 | 0.96 | 0.04 | 0.95 | 56473.49 |

(4b)

| χ2 | **df** | **CFI** | **RMSEA** | **TLI** | **BIC** |
| --- | --- | --- | --- | --- | --- |
| 893.4 | 284 | 0.95 | 0.05 | 0.94 | 56473.49 |

(4c)
