# Supplementary material for: Public communication by research institutes compared across countries and sciences: Building capacity for engagement or competing for visibility?
Source: PLoS One. 2020 Jul 8;15(7):e0235191. doi: 10.1371/journal.pone.0235191 (PMC7343166; doi:10.1371/journal.pone.0235191)
Supplement: S5 Table — (DOCX) [file pone.0235191.s005.docx]

**S5 Table.** Descriptive statistics for main variables.

| **Variable** | **Category** | **Overall Sample (n=2030)** | |
| --- | --- | --- | --- |
|  |  | **n** | **%** |
| Country | Germany | 358 | 18 |
|  | Italy | 366 | 18 |
|  | Portugal | 224 | 11 |
|  | Netherlands | 142 | 7 |
|  | United Kingdom | 188 | 9 |
|  | United States | 262 | 13 |
|  | Brazil | 169 | 8 |
|  | Japan | 321 | 16 |
|  | **Total** | **2030** | **100** |
| OECD research  area | Nat Sci | 512 | 25 |
|  | Eng & Tech | 317 | 16 |
|  | Med & Health | 329 | 16 |
|  | Agri | 107 | 5 |
|  | Soc Sci | 431 | 21 |
|  | Hum | 323 | 16 |
|  | **Total** | **2019** | **100** |
| Comms Staff | Within the unit | 764 | 38 |
|  | Use the central PR offices | 1031 | 51 |
|  | None | 235 | 12 |
|  | **Total** | **2030** | **100** |
| Comms funding | <1% | 681 | 42 |
|  | >=1% | 929 | 58 |
|  | **Total** | **1610** | **100** |
| Comms Policy | No | 849 | 51 |
|  | Yes | 808 | 49 |
|  | **Total** | **1657** | 100 |
| Active researchers | None | 32 | 2 |
|  | < 10% | 287 | 16 |
|  | 10-20% | 402 | 23 |
|  | 20-40% | 348 | 20 |
|  | 40-60% | 339 | 19 |
|  | 60 - 100% | 369 | 21 |
|  | **Total** | **1777** | **100** |
| Research budget | < 100.000 € | 529 | 29 |
|  | 100.000 € - 250.000 € | 286 | 16 |
|  | 250.000 € - 500.000 € | 279 | 15 |
|  | 500.000 € - 1M € | 215 | 12 |
|  | > 1M € | 498 | 28 |
|  | **Total** | **1807** | **100** |
| Size | Less than 20 | 627 | 32 |
|  | 20-79 | 658 | 33 |
|  | 80 or more | 684 | 35 |
|  | **Total** | **1969** | **100** |
