# Supplementary material for: Public communication by research institutes compared across countries and sciences: Building capacity for engagement or competing for visibility?
Source: PLoS One. 2020 Jul 8;15(7):e0235191. doi: 10.1371/journal.pone.0235191 (PMC7343166; doi:10.1371/journal.pone.0235191)
Supplement: S6 Table — Abreviations: Sum of squares (SS); df (degrees of freedom); MS (Mean Square); F statistic (F) and (p (significance value), and Eta (strength of the relationship). (DOCX) [file pone.0235191.s006.docx]

**S6 Table.** Analysis of variance by country and area of research. Abreviations: Sum of squares (SS); df (degrees of freedom); MS (Mean Square); F statistic (F) and (p (significance value), and Eta (strength of the relationship).

|  | **One-way ANOVAS Results** | | |  |  |  |
| --- | --- | --- | --- | --- | --- | --- |
| **Sources of variation** | **SS** | **df** | **MS** | **F** | **p** | **Part Eta** |
| Country |  |  |  |  |  |  |
| Public events | 173630.2 | 7 | 24804.3 | 17.99 | 0.00 | 0.24 |
| Trad news channels | 399694.6 | 7 | 57099.2 | 13.84 | 0.00 | 0.21 |
| New media | 6767406.2 | 7 | 966772.3 | 22.38 | 0.00 | 0.27 |
| Area of research |  |  |  |  |  |  |
| Public events | 18166.8 | 5 | 3633.4 | 2.50 | 0.03 | 0.08 |
| Trad news channels | 59164.0 | 5 | 11832.8 | 2.76 | 0.02 | 0.08 |
| New media | 516933.2 | 5 | 103386.6 | 2.24 | 0.05 | 0.07 |
