# Supplementary material for: Public communication by research institutes compared across countries and sciences: Building capacity for engagement or competing for visibility?
Source: PLoS One. 2020 Jul 8;15(7):e0235191. doi: 10.1371/journal.pone.0235191 (PMC7343166; doi:10.1371/journal.pone.0235191)
Supplement: S8 Table — (DOCX) [file pone.0235191.s008.docx]

**S9 Table**

Summary of hierarchical regression analysis for institutional public communication activity - traditional news media (N=1243)

|  |  | **Model 1** | | |  | **Model 2** | | |
| --- | --- | --- | --- | --- | --- | --- | --- | --- |
|  |  | ***B*** | ***SE B*** | ***β*** |  | ***B*** | ***SE B*** | ***β*** |
| ***Step 1: Context variables*** |  |  |  |  |  |  |  |  |
| Size |  | 0.00 | 0.00 | 0.12*** |  | 0.00 | 0.00 | 0.12*** |
| Research budget |  | 0.07 | 0.01 | 0.27*** |  | 0.06 | 0.01 | 0.21*** |
| Nat Sci |  | -0.05 | 0.05 | -0.05 |  | -0.06 | 0.05 | -0.07 |
| Eng & Techn |  | -0.14 | 0.05 | -0.13** |  | -0.15 | 0.05 | -0.13** |
| Medical & Health sci |  | -0.12 | 0.05 | -0.10* |  | -0.10 | 0.05 | -0.08* |
| Social sci |  | -0.01 | 0.05 | -0.01 |  | -0.06 | 0.05 | -0.06 |
| Humanities |  | 0.01 | 0.05 | 0.01 |  | -0.08 | 0.05 | -0.07 |
| Germany |  | -0.33 | 0.05 | -0.29*** |  | -0.31 | 0.04 | -0.27*** |
| Italy |  | -0.17 | 0.05 | -0.15*** |  | -0.17 | 0.04 | -0.16*** |
| Portugal |  | -0.39 | 0.05 | -0.29*** |  | -0.51 | 0.05 | -0.37*** |
| Netherlands |  | -0.22 | 0.06 | -0.13*** |  | -0.17 | 0.05 | -0.10** |
| United Kingdom |  | -0.32 | 0.05 | -0.23*** |  | -0.29 | 0.05 | -0.21*** |
| United States of America |  | -0.39 | 0.05 | -0.32*** |  | -0.39 | 0.05 | -0.33*** |
| Japan |  | -0.42 | 0.05 | -0.35*** |  | -0.43 | 0.04 | -0.36*** |
| **Step 2: PC-related variables** | | |  |  |  |  |  |  |
| Active researchers |  |  |  |  |  | 0.06 | 0.01 | 0.21*** |
| Policy |  |  |  |  |  | 0.13 | 0.02 | 0.15*** |
| Funding |  |  |  |  |  | 0.04 | 0.01 | 0.11*** |
| Staffing |  |  |  |  |  | 0.14 | 0.02 | 0.16*** |
| (Constant) |  | 0.20 |  |  |  | -0.20 |  |  |
| *Adjusted R^2^* |  | 0.18 | | |  | 0.32 | | |
| *R^2^* change |  |  | | |  | 0.14 | | |
| *F* for change in *R* |  | 20.47*** | | |  | 64.21*** | | |
| **p* < .05 ***p* < .01 ****p* <.001. |  |  |  |  |  |  |  |  |
