# Supplementary material for: Public communication by research institutes compared across countries and sciences: Building capacity for engagement or competing for visibility?
Source: PLoS One. 2020 Jul 8;15(7):e0235191. doi: 10.1371/journal.pone.0235191 (PMC7343166; doi:10.1371/journal.pone.0235191)
Supplement: S9 Table — (DOCX) [file pone.0235191.s009.docx]

**S9 Table**

Summary of hierarchical regression analysis for institutional public communication activity - new media channels (N=1243)

|  | |  | **Model 1** | | | | | | |  | **Model 2** | | | | | | |
| --- | --- | --- | --- | --- | --- | --- | --- | --- | --- | --- | --- | --- | --- | --- | --- | --- | --- |
|  | |  | ***B*** | | | ***SE B*** | | ***β*** | |  | ***B*** | | | ***SE B*** | | ***β*** | |
| **Step 1: Context variables** | |  |  | | |  | |  | |  |  | | |  | |  | |
| Size | | |  | 0.00 | | 0.00 | | 0.06* | | |  | 0.00 | | 0.00 | | 0.05* | |
| Research budget | | |  | 0.02 | | 0.00 | | 0.25*** | | |  | 0.01 | | 0.00 | | 0.20*** | |
| Nat Sci | | |  | 0.00 | | 0.01 | | 0.00 | | |  | -0.01 | | 0.01 | | -0.02 | |
| Eng & Techn | | |  | -0.02 | | 0.01 | | -0.07 | | |  | -0.02 | | 0.01 | | -0.08 | |
| Medical & Health sci | | |  | -0.02 | | 0.01 | | -0.06 | | |  | -0.01 | | 0.01 | | -0.04 | |
| Social sci | | |  | 0.02 | | 0.01 | | 0.07 | | |  | 0.00 | | 0.01 | | 0.01 | |
| Humanities | | |  | 0.03 | | 0.01 | | 0.12* | | |  | 0.01 | | 0.01 | | 0.04 | |
| Germany | | |  | -0.08 | | 0.01 | | -0.30*** | | |  | -0.08 | | 0.01 | | -0.28*** | |
| Italy | | |  | -0.05 | | 0.01 | | -0.20*** | | |  | -0.05 | | 0.01 | | -0.20*** | |
| Portugal | | |  | -0.08 | | 0.01 | | -0.25*** | | |  | -0.11 | | 0.01 | | -0.34*** | |
| Netherlands | | |  | -0.04 | | 0.01 | | -0.09** | | |  | -0.03 | | 0.01 | | -0.07* | |
| United Kingdom | | |  | -0.01 | | 0.01 | | -0.04 | | |  | -0.01 | | 0.01 | | -0.02 | |
| United States of America | | |  | -0.05 | | 0.01 | | -0.18*** | | |  | -0.06 | | 0.01 | | -0.19*** | |
| Japan | | |  | -0.09 | | 0.01 | | -0.32*** | | |  | -0.10 | | 0.01 | | -0.34*** | |
| **Step 2: PC-related variables** | | | | | |  | |  | | |  |  | |  | |  | |
| Active researchers | | |  |  | |  | |  | | |  | 0.01 | | 0.00 | | 0.16*** | |
| Policy | | |  |  | |  | |  | | |  | 0.03 | | 0.01 | | 0.13*** | |
| Funding | | |  |  | |  | |  | | |  | 0.02 | | 0.00 | | 0.18*** | |
| Staffing | | |  |  | |  | |  | | |  | 0.04 | | 0.01 | | 0.16*** | |
| (Constant) | |  | 0.02 | | |  | |  | |  | -0.08 | | |  | |  | |
| *Adjusted R^2^* | |  | 0.17 | | | | | | |  | 0.30 | | | | | | |
| *R^2^* change | |  | 0.18 | | | | | | |  | 0.14 | | | | | | |
| *F* for change in *R* | |  | 18.81*** | | | | | | |  | 61.56*** | | | | | | |
| **p* < .05 ***p* < .01 ****p* <.001. | |  |  | | |  | |  | |  |  | | |  | |  | |
