# Supplementary material for: Public communication by research institutes compared across countries and sciences: Building capacity for engagement or competing for visibility?
Source: PLoS One. 2020 Jul 8;15(7):e0235191. doi: 10.1371/journal.pone.0235191 (PMC7343166; doi:10.1371/journal.pone.0235191)
Supplement: S10 Table — (DOCX) [file pone.0235191.s010.docx]

**S10 Table.** Correlations between main variables.

|  | ‘1’ | ‘2’ | ‘3’ | | ‘4’ | ‘5’ | ‘6’ | ‘7’ | ‘8’ | | | ‘9’ | ‘10’ | |  |  |  |
| --- | --- | --- | --- | --- | --- | --- | --- | --- | --- | --- | --- | --- | --- | --- | --- | --- | --- |
| Events | 1 |  |  | |  |  |  |  |  | | |  |  | |  |  |  |
| Traditional channels | .898** | 1 |  | |  |  |  |  |  | | |  |  | |  |  |  |
| Social Media | .720** | .804** | 1 | |  |  |  |  |  | | |  |  | |  |  |  |
| Size | .142** | .154** | .080** | | 1 |  |  |  |  | | |  |  | |  |  |  |
| Country | -.119** | -.093** | -0.006 | | .085** | 1 |  |  |  | | |  |  | |  |  |  |
| Area research | -.081** | -0.038 | 0.037 | | -0.042 | .051* | 1 |  |  | | |  |  | |  |  |  |
| Research budget | .232** | .258** | .223** | | .145** | -.185** | -.312** | 1 |  | | |  |  | |  |  |  |
| Active Researchers | .210** | .198** | .204** | | -.083** | 0.02 | .174** | -.051* | 1 | | |  |  | |  |  |  |
| Policy | .278** | .275** | .207** | | .090** | -.153** | -0.029 | .085** | .155** | | | 1 |  | |  |  |  |
| Comms Funding | .162** | .166** | .263** | | -0.04 | .139** | .168** | -.113** | .254** | | | .152** | 1 | |  |  |  |
| Comms Staff | .230** | .259** | .257** | | .066** | -.105** | -.064** | .228** | .104** | | | .239** | .153** | |  |  |  |
| ** Correlation is significant at the 0.01 level (2-tailed). | | |  | |  |  |  |  | |  |  | | |  | |  |  |
| * Correlation is significant at the 0.05 level (2-tailed). | | | |  |  |  |  |  | |  |  | | |  | |  |  |
