# Supplementary material for: Public communication by research institutes compared across countries and sciences: Building capacity for engagement or competing for visibility?
Source: PLoS One. 2020 Jul 8;15(7):e0235191. doi: 10.1371/journal.pone.0235191 (PMC7343166; doi:10.1371/journal.pone.0235191)
Supplement: S1 Text — (DOCX) [file pone.0235191.s011.docx]

**Supplementary information text**

**Non-response bias analysis**

The overall response rate was 25% (which assumes that all non-respondents were eligible to participate). Response rates varied across countries, with the Netherlands (NL) (17%) and Brazil (BR) (18%) achieving lower rates; Portugal (PT) (58%), Italy (IT) (33%), United Kingdom (UK) (31%), United States (USA) (24%), Germany (DE) (26%), and Japan (JP) (28%) achieving higher rates.

There is the possibility of nonresponse bias by over-representation of institutes that often engage in public communication (e.g. larger institutions, or sciences more likely to engage in outreach); we investigated whether such non-response bias occurred (1). To assess nonresponse bias we compared early and late respondents. Assuming that late respondents are representative of non-respondents, we can roughly estimate the likelihood of our respondents being significantly different from non-respondents, and weight cases to correct for any possible bias. We categorised the respondents into two groups: early respondents (participation after the launch of the survey and before the third reminder was sent); and late respondents (participation after the third reminder). Using T-tests and Chi square for all variables in the questionnaire, we compared early with late respondents in each country. Overall, we found no significant differences for the tested variables, including our main variables of interest, research area, size, resources (p>0.05). This indicates that there are no differences between institutes that responded earlier or later in the survey, and thus there is no reason to believe that non-respondents would vary significantly.

We could assess potential non-response bias more precisely for the UK and USA samples, given that we had obtained responses from a subsample of initial non-respondents through the supplementary mail survey. Roughly 20% of the supplementary sample returned the questionnaires in both countries. We compared the initial respondents and supplementary respondents on some key variables including ‘policy’, ‘staff’ and ‘funding’ for communications, ‘size’ and ‘scientific area’ and found no significant differences.

Based on this evidence we conclude that non-response would not greatly affect the results in this study; and weighting was then not considered. Results should, nevertheless, be carefully interpreted for findings regarding the NL and BR.

We offer a few possible explanations for the differences in the response rates achieved in the various countries. It is possible that countries such as the UK and the USA, are reaching a saturation point of the topic; in these countries conversations at the academic and political levels have been long standing, and expectations for institutional public engagement is high; see for example, the Research Excellence Framework (REF) for universities, which include impact of research as a main component of evaluation. Also, the fact that some national partners have put somehow more effort into data collection may also in part explain the differences. For example, telephone follow-up was not conducted in the NL nor in BR.

As for size, the overall sample does not seem biased towards larger institutes comprising institutes from a range of sizes including: small (31% less than 20 researchers), medium (34% with 20-80 researchers) and large (35% with more than 80 researchers); thus, providing variety for our analyses. Yet, we find differences in sample sizes across countries with BR and JP having somehow larger institutes. This is possibly a characteristic of the meso-level, which varies slightly across academic and research systems in the surveyed countries, rather than a sample design bias. Size is used as control variable in all our analyses.

Given that our samples include all target populations, the methods used were robust, and the modest response rate is unlikely to invalidate our results, we consider that there the observed institutes offer a good representation the overall samples, and of the institutes in each country. The study represents a novel and significant contribution to our understanding of institutional science communication in these countries.

**Missing value analysis for measures of activities**

We conducted missing value analysis (MVA) to investigate missing value patterns in all variables for events, traditional channels and new media. The percentage of missing values in the items ranged from 2% (in some of the events) to 17% (in some of the new media channels) with the most frequent occurring pattern being 1 missing value per case (around 78-83% of all cases were missing one value). The missing values reflect a systematic rather than random pattern of missingness with higher prevalence of missing values in less frequent activities; a Little’s MCAR test (2) confirms that data are not missing completely at random (p<0.000), and that the missing data is MAR (correlations between variables with missing values and other variables are significant). We repeated the analysis with the DK answers, and found the same patters, and also found strong correlations between the DK and the missing values. The percentage of DK answers, when existed, were below 1%. We then assumed the missingness to be due to no performance of these activities and recoded missing values and DK answers into ‘never’.

1. Sax LJ, Gilmartin SK, Bryant AN. Assessing Response Rates and Nonresponse Bias in Web and Paper Surveys. Res High Educ. 2003;44(4):409–32.

2. Garson GD. Missing Values Analysis and Data Imputation. Asheboro, NC, USA: Statistical Associates Publishing; 2015. 1–26.
