# Supplementary material for: Public communication by research institutes compared across countries and sciences: Building capacity for engagement or competing for visibility?
Source: PLoS One. 2020 Jul 8;15(7):e0235191. doi: 10.1371/journal.pone.0235191 (PMC7343166; doi:10.1371/journal.pone.0235191)
Supplement: S1 File — (DOCX) [file pone.0235191.s012.docx]

UK questionnaire - MORE-PE

Start of Block: Introduction

**Welcome!**
 
This study investigates research units' culture of communication with non-specialist audiences, what we call public engagement. Examples of public engagement activities would be maintaining a website/blog/social media for the public, giving public lectures, participating in public debates, conducting activities for schools, responding to media enquiries, preparing policy papers for decision-makers, and so on. 
 
We will ask you questions about your research unit ${e://Field/RI%20Name}, the resources available for public engagement, and the public engagement activities that your research unit organised/participated over the past 12 months. As some questions may require information you may not have at hand, you might want to take a look at these before you start the survey by downloading them here: [Questionnaire worksheet](https://lse.eu.qualtrics.com/CP/File.php?F=F_bvY0Y5H4XuIJujH)
 
The questionnaire should be completed by a person who is familiar with the research unit, preferably someone involved in the public engagement tasks; completion should take no longer than 20 minutes; some questions may be skipped based on the responses provided. You can also interrupt the survey at any time and return later, your answers will be saved.
  
The study results from an international collaboration involving the London School of Economics and Political Science (UK), ISCTE - Instituto universitário de Lisboa (Portugal), Leiden University (Netherlands), Michigan State University and The University of Texas at Austin (USA), University of Münster (Germany), Observa - Science in Society (Italy), National Sun Yat-Sen University (Taiwan), University of Wroclawski (Poland), Museu da Vida/Casa de Oswaldo Cruz/Fundação Oswaldo Cruz/Fiocruz (Brazil) and the National Graduate Institute for Policy Studies (Japan). For more information on the project and team involved, please visit: [www.more-pe.com](http://www.more-pe.com)
 
For any questions on the language or any technical problems, please do not hesitate to contact [m.entradas@lse.ac.uk](mailto:m.entradas@lse.ac.uk). **The deadline for completion is 28 February 2018.**
 
**We thank you very much** for the time you are taking to complete the questionnaire. Your responses are integral to the success of this study.
 
**Statement of privacy**: All responses are strictly confidential.

| Page Break |  |
| --- | --- |

End of Block: Introduction

Start of Block: Block A (what) - Communication activities by RIs

**We are going to ask you a series of questions about the public engagement activities your research unit participates/organises for non-specialist audiences.**

| 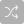 | 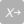 |
| --- | --- |

Q26
**Roughly, how many times in the past 12 months has your research unit engaged in the following events either as organiser or contributor?**

|  | **Never** | **Annually**  (once a year or less) | **Quarterly**  (2-6 times a year) | **Monthly**  (7-20 times a year) | **Weekly**  (> 20 times a year) | **Don't  know** |
| --- | --- | --- | --- | --- | --- | --- |
| Public lectures |  |  |  |  |  |  |
| Exhibitions |  |  |  |  |  |  |
| Open Days, workshops, guided visits and similar events |  |  |  |  |  |  |
| Science Festivals/Science Fairs |  |  |  |  |  |  |
| National Science Week and similar national events |  |  |  |  |  |  |
| Science Cafes and similar formats of public discussions events |  |  |  |  |  |  |
| UNESCO international year, Fame lab, European Researchers' Night and similar international events |  |  |  |  |  |  |
| Deliberative and participatory events on policy-making (*e.g. consensus conferences, citizen juries, consultations, public hearings, policy meetings*) |  |  |  |  |  |  |
| Events organised by private institutions (*e.g. business organisations / industry / corporations*) |  |  |  |  |  |  |
| Talks and workshops at primary/secondary schools |  |  |  |  |  |  |
| Citizen Science projects (*e.g. research projects with shared data collection or collaboration between researchers and non-specialist publics*) |  |  |  |  |  |  |
| Other (*please specify*) |  |  |  |  |  |  |

| Page Break |  |
| --- | --- |

| 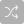 | 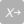 |
| --- | --- |

Q27
**Roughly, how many times in the past 12 months has your research unit engaged, produced or used the following channels to engage with non-specialist audiences?**

|  | **Never** | **Annually**  (once  a year or less) | **Quarterly**  (2-6  times a year) | **Monthly**  (7-20  times a year) | **Weekly**  (> 20  times a year) | **Don't  know** |
| --- | --- | --- | --- | --- | --- | --- |
| Interviews for newspapers |  |  |  |  |  |  |
| Interviews for the radio |  |  |  |  |  |  |
| Interviews for the TV |  |  |  |  |  |  |
| Other TV *(entertainment shows, programs, etc.)* |  |  |  |  |  |  |
| Press conferences |  |  |  |  |  |  |
| Press releases |  |  |  |  |  |  |
| Newsletters |  |  |  |  |  |  |
| Brochures/leaflets/publications for the non-specialist public |  |  |  |  |  |  |
| Articles in magazines, newspapers for the non-specialist public |  |  |  |  |  |  |
| Multimedia/videos/films |  |  |  |  |  |  |
| Popular books |  |  |  |  |  |  |
| Policy papers/briefings on policy issues for industry, politicians, policy-makers |  |  |  |  |  |  |
| Materials for schools *(curriculum, textbooks, etc.)* |  |  |  |  |  |  |
| Other (please specify) |  |  |  |  |  |  |

| Page Break |  |
| --- | --- |

| 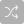 | 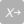 |
| --- | --- |

Q28
**Roughly, how many times in the past 12 months has your research unit used the following social media channels to engage with non-specialist audiences?**

|  | **Never** | **Quarterly**  (2-6  times a year) | **Monthly**  (7-20  times a year) | **Weekly**  (> 20  times a year) | **Daily** | **Don't  know** |
| --- | --- | --- | --- | --- | --- | --- |
| Website updates (*events, content…*) |  |  |  |  |  |  |
| Blogs |  |  |  |  |  |  |
| Facebook |  |  |  |  |  |  |
| Twitter |  |  |  |  |  |  |
| Google+ |  |  |  |  |  |  |
| Instagram |  |  |  |  |  |  |
| YouTube |  |  |  |  |  |  |
| Podcasts |  |  |  |  |  |  |
| Other (*Please specify*) |  |  |  |  |  |  |

| Page Break |  |
| --- | --- |

| 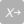 |
| --- |

Q29
**Does your research unit handle the following activities to support public engagement in house or through outsourcing?**

|  | **Within  the unit** | **Within  the host institution** | **Outsourced  to a private company** | **Don't know** |
| --- | --- | --- | --- | --- |
| Formatting/design/layout of materials |  |  |  |  |
| Graphic presentation of data (e.g. charts, graphs, maps) |  |  |  |  |
| Enhanced data visualisation *(e.g. animation, videos)* |  |  |  |  |
| Event organisation/management |  |  |  |  |
| Training in communication/media communication |  |  |  |  |
| Website construction |  |  |  |  |
| Website management |  |  |  |  |
| Other (*please specify*) |  |  |  |  |

End of Block: Block A (what) - Communication activities by RIs

Start of Block: Block B (for Whom) - Audiences addressed by RIs

**We are going to ask you a series of questions about the audiences your research unit engages with.**

| 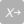 |
| --- |

Q25
**In the table below you will find a list of audiences. How often has your research unit/researchers engaged with each of them in the past 12 months?**

|  | **Never** | **Rarely** | **Occasionally** | **Frequently** | **Very frequently** | **Don't know** |
| --- | --- | --- | --- | --- | --- | --- |
| General public (*whoever might be interested*) |  |  |  |  |  |  |
| Schools |  |  |  |  |  |  |
| Students outside teaching |  |  |  |  |  |  |
| Members of local municipalities/councils/associations |  |  |  |  |  |  |
| Delegates from industry |  |  |  |  |  |  |
| Governments/politicians/policy-makers |  |  |  |  |  |  |
| Non-governmental organisations (NGOs) |  |  |  |  |  |  |
| Media and journalists |  |  |  |  |  |  |
| Other (*Please specify*) |  |  |  |  |  |  |

| Page Break |  |
| --- | --- |

**Now we would like to ask you a few questions about the relations of your research unit with the media.**

| 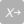 |
| --- |

Q35
**How many times has your research unit received media/journalists enquiries in the last 12 months?**

- Never
- 1 - 2 times
- 3 - 5 times
- 6 - 10 times
- > 10 times
- Don't know

| Page Break |  |
| --- | --- |

| 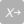 |
| --- |

Q36
**Does your research unit maintain a list of journalists and media contacts?**

- Yes, we have a list/database of journalists and media contacts
- No, we do not have a list but we have personal contacts
- No, we do not have a list/database of journalists and media contacts
- Don't know

| Page Break |  |
| --- | --- |

| 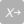 |
| --- |

Q50
**When journalists and the media want to contact researchers at your research unit how do they proceed?**

- They contact our communication/administrative staff first
- Sometimes they contact our communication/administrative staff first, other times they contact the researchers directly
- They contact the researchers directly
- They contact the university/host institution communication, press office directly
- Don't know

| Page Break |  |
| --- | --- |

| 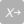 |
| --- |

Q33
**Overall, how successful do you think your public engagement efforts have been in enhancing the activities of your research unit?**

- Very unsuccessful
- Unsuccessful
- Neither successful nor unsuccessful
- Successful
- Very successful

| Page Break |  |
| --- | --- |

| 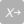 |
| --- |

Q48
**Thinking about the resources your research unit devotes to public engagement (funding, staff, etc.), do you think that your research unit:**

- Should devote less resources to public engagement
- Devotes the right amount of resources to public engagement
- Should devote more resources to public engagement

| Page Break |  |
| --- | --- |

| 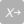 |
| --- |

Q49
**How frequently does your research unit evaluate public engagement activities?

 We evaluate whatever we are doing…**

- Never
- Rarely
- Some of the time
- Most of the time
- Always
- Don't Know

End of Block: Block B (for Whom) - Audiences addressed by RIs

Start of Block: Block C (Who) - PE resources at RIs

**In the following, we are interested in the resources available for public engagement at your research unit. In particular, how many people are involved in public engagement tasks and what funding is available for public engagement initiatives.**

| 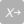 |
| --- |

Q11
**Does your research unit have specialist staff responsible for public engagement activities?**


*Please consider* ***all*** *employees who carry out public engagement tasks as part of their day-to-day responsibilities. This can include staff responsible for maintaining the website, organising public events, supporting researchers in their public engagement work, producing the newsletter, responding to journalists, etc.****For simplicity we will refer to them as 'communication staff****'.*

- Yes, our research unit has its own communication staff
- No, our research unit does not have communication staff but we have access to communication staff within the institution/organisation
- No, our research unit does not have its own communication staff and we do not have access to other communication staff within the institution/organisation

| Page Break |  |
| --- | --- |

| 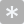 | 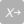 |
| --- | --- |

Q12
**Please indicate how many of the 'communication staff' at your research unit are either exclusively or/and partly dedicated to public engagement tasks.**

 *Please consider****only*** *the communication team and not researchers who conduct their own communication activities.*

Only spend part of their time on public engagement tasks : _______

Are exclusively dedicated to public engagement tasks : _______

Total : ________

| Page Break |  |
| --- | --- |

| 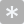 | 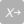 |
| --- | --- |

Q13
**Still thinking about your 'communication staff', please indicate what type of contracts the staff at your research unit have:**


*Please count each person* ***only once****.*

A temporary contract for a specific research project : _______

A temporary contract with the research institute or host institution : _______

A permanent contract with the research institute or host institution : _______

Other type of contract *Please specify* : _______

Total : ________

| Page Break |  |
| --- | --- |

| 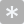 | 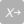 |
| --- | --- |

Q14
**Again thinking about your 'communication staff', please indicate how many of them are full-time/part-time employees.** 


*Please count each person* ***only once.***

Part-time employees : _______

Full-time employees : _______

Total : ________

| Page Break |  |
| --- | --- |

| 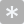 | 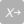 |
| --- | --- |

Q17
**Finally, considering only those of the 'communication staff' who are not exclusively dedicated to public engagement tasks, in your judgment, on average, how many hours per week do they spend on such tasks?**

Half day per week or less : _______

1 day per week : _______

2-3 days per week : _______

4 or more days per week : _______

Total : ________

| Page Break |  |
| --- | --- |

| 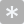 | 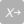 |
| --- | --- |

Q15
**Still thinking about the 'communication staff' at your unit, please indicate how many have an education degree in the following research areas:**


*Please count each person* ***only once****.*

Natural Sciences : _______

Engineering and Technology : _______

Medical and Health Sciences : _______

Agricultural Sciences : _______

Social Sciences : _______

Humanities : _______

No university degree : _______

Total : ________

| Page Break |  |
| --- | --- |

| 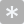 | 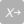 |
| --- | --- |

Q47
**Which of the following most closely matches the previous professional experiences of the 'communication staff' at your research unit?**


*Please count each person* ***only once****, based on their most relevant previous job.*

No previous professional experience : _______

Worked as researchers : _______

Worked as teachers : _______

Worked as marketing/public relations/communication professionals : _______

Worked as design/multimedia professionals : _______

Worked as journalists/science writers : _______

Worked as administrative professionals : _______

Worked as project/finance/human resources officers/managers : _______

Other *(please specify)* : _______

Total : ________

| Page Break |  |
| --- | --- |

| 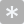 | 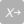 |
| --- | --- |

Q16
**How many of the 'communication staff' at your research unit have a background and/or training in communication (e.g. media, digital, corporate communications, science communication)?**


*Please count each person* ***only once*** *based on their highest degree of specialization.*

No formal training in communication : _______

Attended workshops/short courses in communication : _______

Undergraduate degree related to communication : _______

Post-graduate degree related to communication (post-graduation course, masters or PhD) : _______

Total : ________

| Page Break |  |
| --- | --- |

| 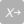 |
| --- |

Q18
**For how long has your research unit been carrying out public engagement activities for non-specialist audiences?**

- Less than 1 year
- Between 1 - 5 years
- Between 5 - 10 years
- More than 10 years
- Don't know

| Page Break |  |
| --- | --- |

| 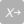 |
| --- |

Q19
**Over the last 5 years, the total number of public engagement activities for non-specialist audiences at your research unit has:**


*If the communication structure at your unit was created less than 5 years ago, please consider the period of time it was created.*

- Decreased
- Stayed the same
- Increased
- Don't know

| Page Break |  |
| --- | --- |

| 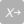 |
| --- |

Q52
**Overall how frequently does the 'communication  staff' at your research unit conduct the following?**

|  | **Never** | **Rarely** | **Occasionally** | **Frequently** | **Very frequently** | **Don't know** |
| --- | --- | --- | --- | --- | --- | --- |
| Decide on public engagement policies with the leadership of the research unit |  |  |  |  |  |  |
| Create/propose public engagement action plans to the leadership of the research unit |  |  |  |  |  |  |
| Motivate researchers to get involved in public engagement events |  |  |  |  |  |  |
| Intervene in moments of institutional reputation 'crisis' |  |  |  |  |  |  |
| Compose/edit/print communication materials *(press releases, newsletters, leaflets, powerpoint presentations, etc.)* |  |  |  |  |  |  |
| Compose/edit/stream audiovisual materials *(photographs, videos, etc.)* |  |  |  |  |  |  |
| Manage the website and online communication of the research unit *(social media channels such as Twitter, Facebook, etc.)* |  |  |  |  |  |  |
| Organise public events *(Open days, Science Weeks, talks, workshops, etc.)* |  |  |  |  |  |  |
| Organise/offer communication training for researchers *(Public speaking, Media training, etc.)* |  |  |  |  |  |  |
| Assist researchers on planning/completing research grant applications |  |  |  |  |  |  |

| Page Break |  |
| --- | --- |

| 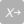 |
| --- |

Q20
**Please estimate the percentage of the annual budget spent in the last 12 months on the public engagement efforts of your research unit. This can include actions such as maintenance of the website, printing of brochures, organisation of public events, etc. Please do not consider salaries of the 'communication staff'.**

- None
- < 1%
- 1 - 5%
- 5 - 10%
- > 10%
- Don't Know

| Page Break |  |
| --- | --- |

| 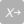 |
| --- |

Q54 **Could you please estimate the average research income of your research unit over the last 3 years [(2014+2015+2016) / 3]**

- < £100,000
- £100,000 - £250,000
- £250,000 - £500,000
- £500,000 - £1M
- > £1M

| Page Break |  |
| --- | --- |

| 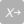 |
| --- |

Q21
**How is your research unit funded? Roughly, what percentage of funds are sourced from recurrent core institutional funding and what percentage comes from external projects' funding?**

▼ 100% internal funds ... Don't Know

| Page Break |  |
| --- | --- |

| 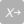 |
| --- |

Q22
**Please tell us whether the following statements about the commitment of your research unit to public engagement are true or false:**

|  | **False** | **True** | **Don't know** |
| --- | --- | --- | --- |
| We have a public engagement policy |  |  |  |
| We have public engagement action plans |  |  |  |
| We expect our researchers to be involved in communication with the public |  |  |  |
| Our communication efforts respond to the national policies on public engagement |  |  |  |
| We have neither plans nor a policy for public engagement but we nevertheless engage with the public |  |  |  |

| Page Break |  |
| --- | --- |

| 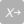 |
| --- |

Q5
**How many people work at your research unit? Please consider all researchers, post-doctoral fellows and PhD students and technicians; do not count administrative staff.**

_______ researchers work at our research unit

| Page Break |  |
| --- | --- |

| 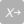 |
| --- |

Q23
**Roughly, in the last 12 months what percentage of researchers at your research unit including PhD students, fellows, visitors, and regular research staff, took part in public engagement activities? This can include activities such as public lectures, public debates, activities at schools, National Science Week, etc.**

- None
- < 10%
- 10 - 20%
- 20 - 40%
- 40 - 60%
- 60 - 100%
- Don't know

| Page Break |  |
| --- | --- |

| 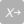 |
| --- |

Q24
**For those researchers at your unit who do not engage in public engagement activities, what do you think is discouraging them to do it?**

|  | **Definitely not true** | **Unlikely true** | **Likely true** | **Very likely true** | **Don't know** |
| --- | --- | --- | --- | --- | --- |
| They are not enthusiastic about communicating their work to general audiences |  |  |  |  |  |
| They do not perceive public engagement as their everyday work/responsibility |  |  |  |  |  |
| They do not perceive public engagement as contributing to the progress of their careers |  |  |  |  |  |
| They do not have time for it |  |  |  |  |  |
| They are not rewarded for their public engagement work |  |  |  |  |  |
| They lack institutional support for doing it *(e.g. staff, training, funding)* |  |  |  |  |  |
| They see communication as the responsibility of the communication staff rather than their own |  |  |  |  |  |
| They think the public is not interested in the research they do |  |  |  |  |  |
| They feel they are not good at it |  |  |  |  |  |
| Other reason *(please specify)* |  |  |  |  |  |

| Page Break |  |
| --- | --- |

| 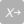 |
| --- |

Q41
**Lastly, what percentage of your unit's annual budget do you think should be allocated to your public engagement efforts?**

- ⊗None
- ⊗< 1%
- ⊗1 - 5%
- 5 - 10%
- >10%
- Don't know

End of Block: Block C (Who) - PE resources at RIs

Start of Block: Block D (Why) - Rationales, perceptions and ethos

**Now we would like to ask you some questions about your unit's rationales for communication, perceptions of media coverage and the public, and outcomes of public engagement.**

| 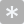 |
| --- |

Q10
**What would you say is the most important, second most important and least important reason for your research unit to undertake communication with non-specialist audiences?**
 
Please drag the selected statements and drop them in the box of your choice

| MOST important reason (please drag only one item) | SECOND MOST important reason (please drag only one item) | LEAST important reason (please drag only one item) |
| --- | --- | --- |
| ______ We aim to respond to the policy/mission of our host institution/university | ______ We aim to respond to the policy/mission of our host institution/university | ______ We aim to respond to the policy/mission of our host institution/university |
| ______ We aim to respond to the policy of our funding bodies | ______ We aim to respond to the policy of our funding bodies | ______ We aim to respond to the policy of our funding bodies |
| ______ We aim to respond to national policies of public engagement | ______ We aim to respond to national policies of public engagement | ______ We aim to respond to national policies of public engagement |
| ______ We want to raise our research profile | ______ We want to raise our research profile | ______ We want to raise our research profile |
| ______ We want to attract funding | ______ We want to attract funding | ______ We want to attract funding |
| ______ We want to get public support for the research we do | ______ We want to get public support for the research we do | ______ We want to get public support for the research we do |
| ______ We want to disseminate our research to the public | ______ We want to disseminate our research to the public | ______ We want to disseminate our research to the public |
| ______ We want to listen and involve the public in our research | ______ We want to listen and involve the public in our research | ______ We want to listen and involve the public in our research |
| ______ We aim to recruit new generations of scientists | ______ We aim to recruit new generations of scientists | ______ We aim to recruit new generations of scientists |
| ______ Other <em>(Please specify)</em> | ______ Other <em>(Please specify)</em> | ______ Other <em>(Please specify)</em> |

| Page Break |  |
| --- | --- |

| 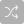 | 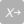 |
| --- | --- |

Q30
**People look to science for different reasons. Please indicate how likely your research unit is to use the following criteria when deciding what research results to communicate with non-specialist audiences.**
 
**We communicate what is...**

|  | **Very  unlikely** | **Unlikely** | **Neither likely  nor unlikely** | **Likely** | **Very  likely** | **Don't  know** |
| --- | --- | --- | --- | --- | --- | --- |
| Relevant to daily life |  |  |  |  |  |  |
| Relevant to current debates |  |  |  |  |  |  |
| What people should know |  |  |  |  |  |  |
| Innovative/new developments and findings |  |  |  |  |  |  |
| Entertaining and interesting |  |  |  |  |  |  |
| Other reason *(Please specify)* |  |  |  |  |  |  |

| Page Break |  |
| --- | --- |

| 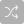 | 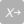 |
| --- | --- |

Q37
**To what extent do you agree or disagree with the following statements concerning media coverage of the research conducted at your research unit?**

|  | **Strongly  disagree** | **Disagree** | **Neither  agree nor  disagree** | **Agree** | **Strongly  agree** | **Don't know** |
| --- | --- | --- | --- | --- | --- | --- |
| Visibility in the media of the research conducted at our unit is important |  |  |  |  |  |  |
| Media should give more attention to the research conducted at our unit |  |  |  |  |  |  |
| Journalists have reported badly about our work |  |  |  |  |  |  |
| The research we do is of little interest to journalists |  |  |  |  |  |  |
| To maintain media relations is not our task |  |  |  |  |  |  |

| Page Break |  |
| --- | --- |

| 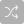 | 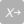 |
| --- | --- |

Q39
**The following statements express opinions about the public. To what extent do you agree or disagree with each statement?**

|  | **Strongly disagree** | **Disagree** | **Neither agree nor disagree** | **Agree** | **Strongly agree** | **Don't know** |
| --- | --- | --- | --- | --- | --- | --- |
| The public is not interested in the research conducted at our unit |  |  |  |  |  |  |
| The public is not eager to learn about science |  |  |  |  |  |  |
| The public is interested in a limited range of research topics such as dinosaurs, dolphins and disasters |  |  |  |  |  |  |
| The public wants to contribute to science |  |  |  |  |  |  |
| We cannot expect a large public to take interest in the research we do |  |  |  |  |  |  |
| If the public knows more about our research, they will be more likely to support it |  |  |  |  |  |  |
| The public does not need to understand the full picture, we explain what we think is appropriate |  |  |  |  |  |  |
| The public needs to be educated by those who are knowledgeable |  |  |  |  |  |  |

| Page Break |  |
| --- | --- |

| 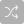 | 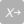 |
| --- | --- |

Q40
**The following statements express opinions about the public. To what extent do you agree or disagree with each statement? *(cont.)***

|  | **Strongly disagree** | **Disagree** | **Neither agree nor disagree** | **Agree** | **Strongly agree** | **Don't know** |
| --- | --- | --- | --- | --- | --- | --- |
| We communicate with the public very selectively to avoid trouble |  |  |  |  |  |  |
| We would like the public to become more actively involved in decisions about the research conducted at our research unit |  |  |  |  |  |  |
| We would like the public to become more involved in discussing the implications of the research we do, but not necessarily in decisions about our research directions |  |  |  |  |  |  |
| The public trusts science and scientists |  |  |  |  |  |  |
| The public does not need to be scientifically literate to discuss the implications of our research |  |  |  |  |  |  |

| Page Break |  |
| --- | --- |

End of Block: Block D (Why) - Rationales, perceptions and ethos

Start of Block: Block E - General info on RIs/Respondents

**In the following we would like to ask you some general questions about your research unit.**

Q3
**When was your research unit founded? (Please provide the year or an estimate)**

Year

▼ Don't know ... 1800

| Page Break |  |
| --- | --- |

| 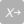 |
| --- |

Q4
**Which of the following best describes the split between research and teaching at your unit?**

- We only do research
- We do more research than teaching
- We do more teaching than research
- Research and teaching are equally balanced

| Page Break |  |
| --- | --- |

Q7
**Please select from the list below the main scientific area of your unit and the main discipline.**

**Main scientific area**

**Main scientific discipline**

▼ Natural Sciences ... Humanities ~ Multidisciplinary

| Page Break |  |
| --- | --- |

**To end the questionnaire, we would like to ask you a few questions about yourself. Please note that all information will be kept confidential.**

| 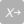 |
| --- |

Q42
**Which of the following most closely matches your job title?**

- Researcher
- Management/Administrative staff
- Communication staff
- Unit's Director/Coordinator/Head of institute
- PhD student
- Other *(Please specify)* ________________________________________________

| Page Break |  |
| --- | --- |

Q43
**For how long have you been working in this role?**

Years (roughly)

▼ 1 ... 50

| Page Break |  |
| --- | --- |

| 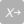 |
| --- |

Q51
**How many people were involved in answering this survey?**

- Just me
- Me and other people *(Please specify how many)* ________________________________________________

| Page Break |  |
| --- | --- |

| 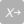 |
| --- |

Q53
**Which of the following most closely matches the job titles of the people who helped you answering the survey? Pick all relevant**

- Researcher
- Management/Administrative staff
- Communication staff
- Unit´s Director/Coordinator/Head of institute
- PhD student
- Other (Please specify) ________________________________________________

| Page Break |  |
| --- | --- |

Q00 **If you have any comments to the survey, please express them here**

________________________________________________________________

________________________________________________________________

________________________________________________________________

________________________________________________________________

________________________________________________________________

End of Block: Block E - General info on RIs/Respondents
